# A consensus score to combine inferences from multiple centres

**DOI:** 10.1007/s00335-023-09993-0

**Published:** 2023-05-08

**Authors:** Hamed Haselimashhadi, Kolawole Babalola, Robert Wilson, Tudor Groza, Violeta Muñoz-Fuentes

**Affiliations:** European Bioinformatics Institute, European Molecular Biology Laboratory, Hinxton, UK

## Abstract

Experiments in which data are collected by multiple independent resources, including multicentre data, different laboratories within the same centre or with different operators, are challenging in design, data collection and interpretation. Indeed, inconsistent results across the resources are possible. In this paper, we propose a statistical solution for the problem of multi-resource consensus inferences when statistical results from different resources show variation in magnitude, directionality, and significance. Our proposed method allows combining the corrected p-values, effect sizes and the total number of centres into a global consensus score. We apply this method to obtain a consensus score for data collected by the International Mouse Phenotyping Consortium (IMPC) across 11 centres. We show the application of this method to detect sexual dimorphism in haematological data and discuss the suitability of the methodology.

**Supplementary Information:**

The online version contains supplementary material available at 10.1007/s00335-023-09993-0.

## Introduction

Measuring response to a treatment based on data collected from multiple resources, such as multicentre clinical trials or animal experiments, benefits from (1) lower noise level, because results are not strongly resource-dependent (Karp et al. 2014), and (2) effectiveness, because they apply to a broader population (Rashid et al. 2012; Karp et al. 2017). In these experiments, obtaining a global consensus in the statistical inference across resources is desired. However, even in highly controlled experiments, it is not always possible to control for all sources of variation across all resources. This makes aggregating statistical results from multiple resources challenging because the results may be vulnerable to biases, which lead to inconsistent inferences. The design of the study, sample size, power of the analysis, variation across centres or over time (Haselimashhadi et al. 2020a) and unknown errors are examples of factors that pose a challenge to obtaining a global statistical conclusion across resources (Chung et al. 2010; Hu et al. 2022; Knatterud et al. 1998). Other confounders are the equipment that is used to perform the measurements in different resources (e.g., centres, laboratories, etc.), the level of experience of the staff and more complex environmental factors that typically arise in animal tests, such as diet, litter, handling, circadian rhythm, housing and husbandry. Therefore, in multi-resource experiments, it is crucial to control for as many variables as possible, to be able to reach global agreements (Haselimashhadi et al. 2020a; Chung et al. 2010; Chalmers and Clarke 2004; Hogg 1991). Table 1 shows some examples of possible outcomes when an experiment is conducted in 4 centres.

**Table 1:** Examples of possible outcomes when a global inference from statistical results obtained from multiple centres is desired

Scenario	Setup	Inference
1	All centres achieve statistically significant results	Global consensus
2	2 centres achieve statistically significant results 2 centres did not achieve statistically significant results	Not clear
3	2 centres achieve statistically significant results 2 centres did not achieve statistically significant results but 1 of them has a borderline p-value	Not clear
4	2 centres strongly achieved statistically significant results 2 centres did not achieve statistically significant results with p-values strongly diverting from the significant level	Not clear
5	All centres achieved statistically significant results but 2 in the positive and 2 in the opposite direction	Not clear
6	2 centres achieved statistically significant results 1 centre achieved statistically significant results in an opposite direction 1 centre did not achieve/borderline statistically significant results	Not clear

In this table, we focus on the treatment effect size and *p* values from centres and assume that the experiment is highly controlled and conducted by 4 centres (e.g. laboratories)

In this paper, we present a methodological approach which seeks to find a solution to the problem of multi-resource consensus with a focus on multicentre experiments. The proposed method allows calculating a global consensus score for the effect of interest (i.e., research questions, e.g., genotype, sexual dimorphism, bodyweight effect) in multicentre studies. The method takes into consideration the number of centres where the test of interest is performed at, the direction and magnitude of the effect size and the significance level obtained from individual centres and combines the values into a global consensus score. We apply our method to data obtained by the International Mouse Phenotyping Consortium (IMPC), a transnational multicentre endeavour that screens the phenotypes of single-gene knockout mouse lines and wild-type mice to understand gene function (Koscielny et al. 2014).

## Method

There are several approaches typically used to aggregate inferences from multicentre data. Among them, three major methods involve adjusting for centres using fixed and random models; or analysing each centre separately and then combining the results using meta-analyses (Rashid et al. 2012; Basagaña et al. 2018; Burke et al. 2017; Bowden et al. 2011; Stewart et al.^2012^). Other methods are utilising group decision-making processes, such as the DELPHI method (Ven and Delbecq 2017; Dalkey and Helmer 1963); or using a simple majority rule criteria, such as *all centres agree* versus *at least one centre disagree*; or employing simple statistics or probabilistic criteria, such as *more than half/mean/median centres/results agree* or simple statistical tests such as T-test or ANOVA (Mlecnik et al. 2020). Latter approaches may suffer from insufficient power, individual bias (such as misjudgements or making decisions based on insufficient information) and may have strong underlying assumptions as well as require a large M, the total number of centres, to converge to the true inference (Rashid et al. 2012; Using the Delphi method 2022).

Here we propose an alternative approach which combines the corrected p-values (q-values), which we obtained using the FDR (Controlling the False Discovery Rate 2022; Wright 1992; Hochberg 1988), and the effect sizes from individual centres and compares them with a set of expected values as below:

1 $$\mathrm{Consensus score }\left(\mathrm{s}\right)= \left\{\begin{array}{c}\frac{{\sum }_{i}\left({q}_{i}\times \sqrt{\left|{\rho }_{i}\right|}\right)}{\overline{M}{ }^{2}\times \widehat{q}\times \sqrt{\widehat{\rho }}}\times Max\left(\frac{M}{2},\overline{M }\right) , \overline{M }\times P>c\\ 1 \,\,\,\,\,\,\,\,\,\,\,\ , o.w\end{array}\right.$$

where *i* = 1, 2,…,*M* represents the i^th^ centre from a total of $M$ centres, $\overline{M }$ the total number of centres where the test is performed at ($M$ is not necessarily equivalent to $\overline{M }$ in multicentre multi-test studies where the aim is to compare several measurements across centres while fixing the number of centres), ${q}_{i}$ the corrected *p* value (q-value) from the statistical test performed in centre $i$ for the effect of interest (e.g. sex, genotype, body weight effect, etc.), ${\rho }_{i}$ the estimated standardised effect size from the statistical test that is performed in centre $i$, such as Cohen’s $d$ effect size (Ellis 2010) and $P=|{\sum }_{i}\mathrm{Sign}\left({\rho }_{i}\right)/\overline{M }|$ is a penalty term to control for the directionality of the results, and the $\mathrm{Sign}\left(\rho \right)$ is the sign function defined by

$$\mathrm{Sign}\left(\rho \right)=\left\{\begin{array}{c}1 \rho >0\\ 0 \rho =0\\ -1 \rho <0\end{array}\right..$$

**Fig. 1:**
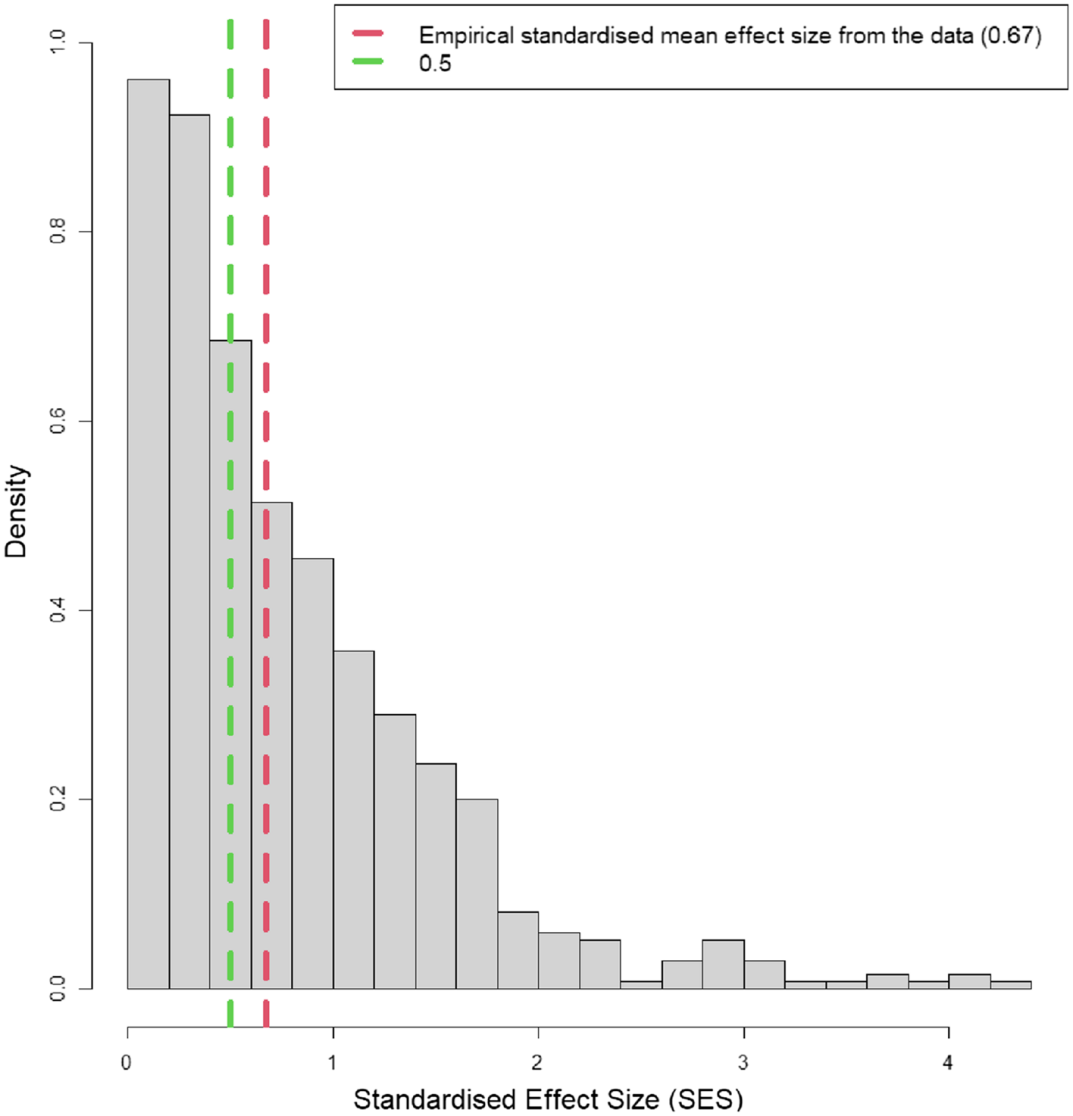
The distribution of the standardised effect sizes (SES) for the IMPC haematological traits. The empirical 10% trimmed mean SES (dashed red line) is 0.67 and the recommended value for the expected effect size ($\widehat{\uprho }$) is 0.5 (dash green line)

Finally, $c$*,* $\widehat{q}$ and $\widehat{\rho }$ are the minimum required number of centres for the analysis, the expected q-value and effect size from the prior information, respectively. We recommend $c=3$, $\widehat{q}=0.05$ and moderate expected effect size $\widehat{\rho }=0.5$ (Karp et al. 2017; Sullivan and Feinn 2012; Sawilowsky 2009) as the preliminary values for high-throughput experiments, such as in the IMPC. We stress that the choice of these parameters should be based on prior information. The choice of the expected q-value or the minimum number of required centres should take into account the context of the study, the sensitivity of the results or expert knowledge in the field; the expected effect size can be set from prior studies, simulations or empirical results, as we show in Fig. 1. This figure shows the distribution of the standardised effect sizes for the IMPC haematological traits and empirical mean ($10\%$ trimmed) from the data and the recommended expected effect size, $\widehat{\rho }$ = 0.5. We further assume that (1) there is no unusual temporal variation in the data (Supplementary Fig. 1), (2) the statistical tests are consistent and sufficiently powerful and adequate for the data under study, (3) the method to adjust the p-values is adequate (e.g. FDR); and (4) the effect sizes are estimated from the normalised data. Here normalising data refers to performing the statistical analysis on the standardised data as below:

$$\mathrm{standardised data for centre} i=\frac{{x}_{i}-{\mu }_{xi}}{{\sigma }_{xi}}$$

where ${x}_{i}$, ${\mu }_{xi}$ and ${\sigma }_{xi}$ are the raw values, mean and standard deviation of the data from centre $i$ respectively. The resulting scores from Eq. 1 range in the $\left(0,+\infty \right)$ interval and the agreement of the multicentre statistical results can be evaluated by using $-\mathrm{log}\left(\mathrm{s}\right)$ so that

$$\left\{\begin{array}{c}Consensus across centres if -\mathrm{log}\left(\mathrm{s}\right)>0 \\ Not enough consensus across centres if -\mathrm{log}\left(\mathrm{s}\right)\le 0\end{array}.\right.$$

The magnitude of $-\mathrm{log}\left(\mathrm{s}\right)$ from Eq. 1 is not bounded. As a result, a larger value in the positive (or negative) direction reflects a stronger agreement (or lack of agreement) among resources. For the special case where $-\mathrm{log}\left(\mathrm{s}\right)=0$, one can conclude that either there is not enough information in the data to calculate the scores or there is not enough agreement across centres. Throughout this paper, we use the term “not enough agreement” in contrast to “disagreement” to emphasize the difference between strong detection of consensus and not finding enough evidence to establish consensus among centres. Table 2 shows several scenarios as well as the inferences from the scores in Eq. 1. This table shows that the most ambiguous scenario happened when all centres achieved the same effect size and q-value to the expected values (scenario 2) or the centre achieved a range of opposite (in sign) effects so that $M\times P\le 3$ (scenario 3). Because ${\mathrm{q}}_{\mathrm{i}}$ and ${\mathrm{p}}_{\mathrm{i}}$ are continuous real values, ${\mathrm{q}}_{\mathrm{i}},|{\mathrm{p}}_{\mathrm{i}}|\in [0,\infty )$, scenario 3 happens with an extremely low chance that can be safely neglected.

**Table 2:** The demonstration of the scores calculated from Eq. 1 in a set of scenarios with 3 or more centres when the proposed scoring method in Eq. 1 leads to different values and inferences

Scenario	Setup	Score [-log(score)]	Inference
1	Less than 3 centres	–	Does not reach the minimum requirement for the analysis
2	1. There are $M>3$ centres 2. The arrangement of effect sizes is so that $M\times P\le 3$	$S=1 \left[0\right]$	There is not enough information in the data to make the inference
3	1. More than 3 centres 2. All centres have the same q-value equal to the expected q-value (e.g. 0.05) and effect size equal to the expected effect sizes (e.g. 0.5)	The nominator and denominator cancel each other and consequently $S=1 \left[0\right]$	Not enough agreement between centres
4	1. More than 3 centres 2. q-values and effect sizes are greater than expected values	The nominator is greater than the denominator and $S>1 \left[>0\right]$	Not enough agreement between centres
5	1. More than 3 centres 2. q-values and effect sizes are all less than expected values	The nominator is less than the denominator and $S<1 \left[<0\right]$	Full agreement between centres
6	1. More than 3 centres 2. q-values and effect sizes are distributed so that the mean $\frac{{\sum }_{i}\left({q}_{i}\times \sqrt{\left|{\rho }_{i}\right|}\right)}{\overline{M} }$ is less than the expected mean in the denominator	The nominator is less than the denominator and $S<1 \left[<0\right]$	Agreement between centres
7	1. More than 3 centres 2. q-values and effect sizes are distributed so that the mean $\frac{{\sum }_{i}\left({q}_{i}\times \sqrt{\left|{\rho }_{i}\right|}\right)}{\overline{M} }$ is greater than the expected mean (in the denominator	The nominator is greater than the denominator and $S>1 \left[>0\right]$	Not enough agreement between centres
8	1. More than 3 centres 2. Effect sizes are distributed non-uniformly between centres so that some centres detect high effect sizes (e.g. > 1.5) and some very small effect sizes (e.g. < 0.5)	Because the decision is made based on the square root of effect sizes and because of the mathematical properties of the square root function below, the method is robust to the variations and the scores remain valid $$\left\{\begin{array}{c}\sqrt{x}>x if x<1\\ \sqrt{x}\le x if x\ge 1\end{array}\right.$$	Inference based on the final value of $S$

Scenarios 2 and 3 lead directly to a score of $1 \left(\mathit{log}\left(s\right)=0\right)$ with two different inferences: (i) there is not enough information in the data to make the inference; or (ii) not enough agreement between centres. Because ${q}_{i}$ and ${p}_{i}$ are continuous real values, ${q}_{i},|{p}_{i}|\in [0,\infty )$, In practice, scenario 3 happens with an extremely low chance and can be safely ignored. The first scenario should be detected in a pre-processing step

## Results

In this section, we show the application of the proposed scoring method along with two methods from the literature, precisely global consensus and metadata analysis, to identify sexual dimorphism in the IMPC haematological data collected from wild-type (WT) mice, with an average age of 16–18 weeks, over a 3-year period from 1st January 2018 to 31st December 2020, with a minimum required threshold of 50 mice per sex. Our choice of data is inspired by the importance of the haematology parameters reflecting overall health. The data used in this study can be accessed via the IMPC web portal under the URL www.mousephenotype.org (data release 15.1—October 2021).

The IMPC is a global effort aiming to generate and characterise knockout mouse lines for every protein-coding gene in mice (Dickinson et al. 2016; Bradley et al. 2012; Brown and Moore 2012; Hrabě de Angelis et al. 2015). The IMPC data are collected from several independent centres worldwide (Koscielny et al. 2014). Every centre contributes to the data collection by adhering to a set of standardised phenotype assays defined in the International Mouse Phenotyping Resource of Standardised Screens (IMPReSS—www. mousephenotype.org/impress). Although all centres follow the same Standard Operating Procedures (SOPs), there may be unavoidable or necessary variations in the implementation of the experiments (such as mouse age or time of the day when the test is performed), equipment (such as manufacture, model and kits) as well as the level of expertise and experience of staff (experimenter effect), in addition to variations in inbred mouse strain (Table 3) (Bryant et al. 2008). This may lead to differing results across centres, which makes a global inference from the results challenging.

**Table 3:** Mouse strains that are used by the IMPC centres for the haematological data collected from 1st January 2018 to 31st December 2020

IMPC centre	BCM	CCP-IMG	HMGU	ICS	JAX	KMPC	MRC Harwell	RBRC	TCP	UC Davis	WTSI
Mouse strain											
C57BL/6N	✓	–	–	✓	–	–	–	–	–	–	✓
C57BL/6NCrl	–	✓	✓	–	–	–	–	–	✓	✓	–
C57BL/6NJ	–	–	–	–	✓	–	–	–	–	–	–
C57BL/6NJcl	–	–	–	–	–	–	–	✓	–	–	–
C57BL/6NTac	–	–	–	–	–	✓	✓	–	–	–	–

### IMPC haematology

**Fig. 2:**
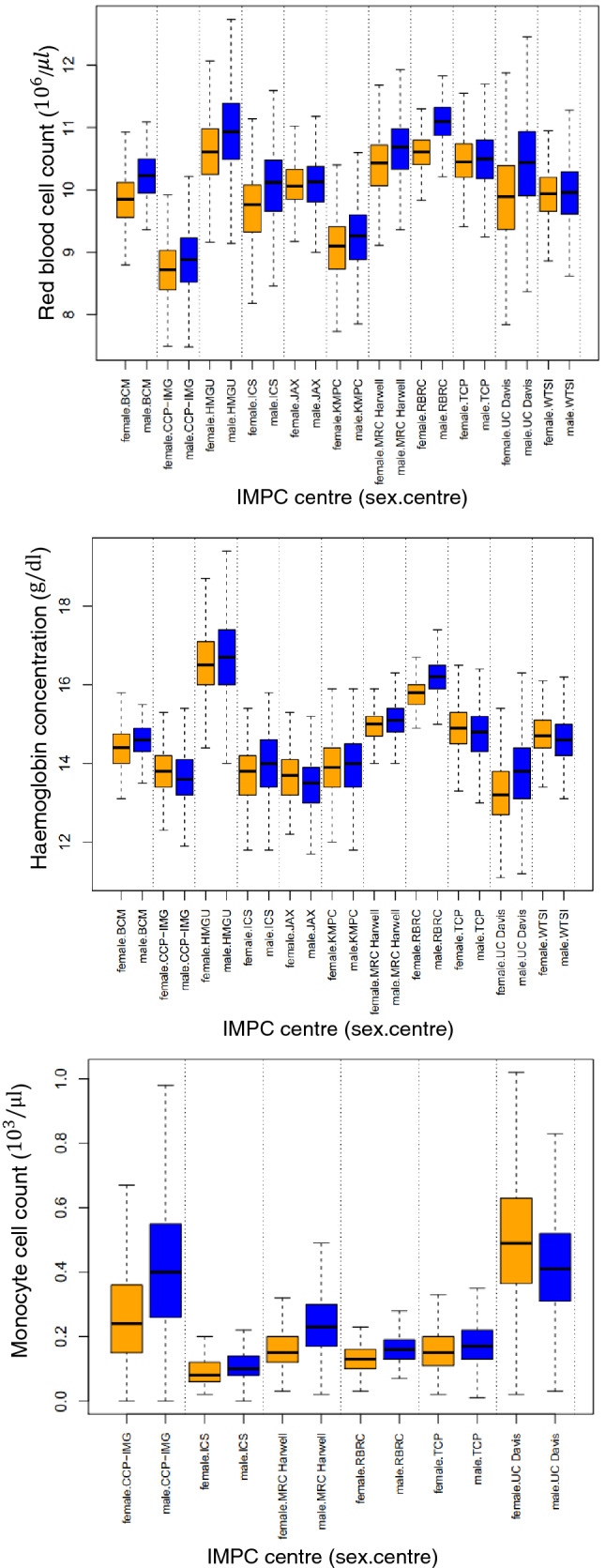
The distribution of red blood cell counts (top), the haemoglobin concentration (middle) and monocyte cell counts (bottom) for wild-type mice from the IMPC, split by sex and phenotyping centre. The orange and blue represent females and males, respectively. The consensus score for the red blood and monocyte cell count traits are respectively $-\mathrm{log}\left(\mathrm{s}\right)=0.30$ and 2.28 which implies a global agreement across IMPC centres in identifying sexual dimorphism; the sign of the average effect size indicates whether males (positive) or females (negative) present higher values (males in this case, see Table 2). In contrast, the consensus score for the haemoglobin concentration trait is $-\mathrm{log}\left(\mathrm{s}\right)=0$, which implies lack of agreement among the IMPC centres to detect sexual dimorphism for this parameter

The IMPC haematology procedure encapsulates 22 measurements of blood properties such as counts and concentrations (white blood cell count, red blood cell count, haemoglobin concentration, platelet counts, etc.), as well as additional and derived haematological parameters (haematocrit, mean red blood cell volume, mean red blood cell haemoglobin, mean red blood cell haemoglobin concentration, etc.). Figure 2 (top) shows red blood cell counts, (middle) the haemoglobin concentration and (bottom) the monocyte cell counts collected by IMPC centres. The shifts in the means are most likely due to differences in the equipment used to take the measurements and can be removed by normalising the data. The top plot shows consistently higher red blood cell counts in males than females across centres, whereas there is not a clear pattern for the haemoglobin concentration. For the monocyte counts, males present consistently higher values, except for one centre, which shows the opposite.

### Consensus score

**Fig. 3:**
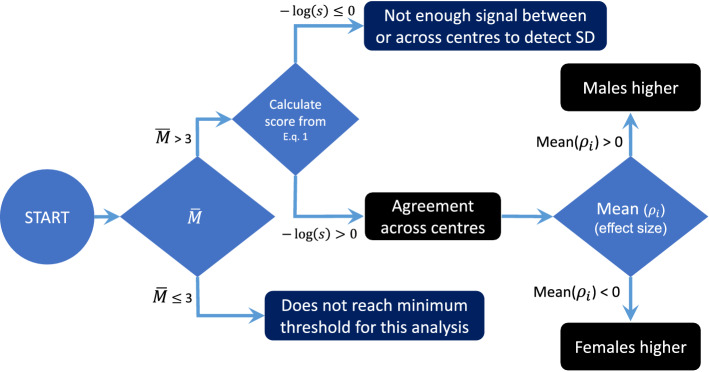
Flowchart showing the logic behind the scoring method to obtain a consensus global inference from multicentre results. The first step involves examining the number of centres performing the test; when there are more than 3 centres, the consensus score is calculated. Provided $-\mathit{log}\left(s\right)>0$, a multicentre consensus signal is established (accepted) and the direction of sexual dimorphism based on the sign of the average effect sizes is reported

In line with (Karp et al. 2017), the sexual dimorphism effect is tested for all 22 haematology traits, independently for WT mice from individual centres, corresponding to the same mouse strain and metadata group split. We used a linear mixed model described in Haselimashhadi et al. 2020b; Gałecki and Burzykowski 2013) and implemented in the software R (Team RC-VRC 2013) and packages OpenStats (Mashhadi 2023). As in Karp et al. (2017), $Sex$ and $Body Weight$ in the fixed effect terms

$$Response=Sex+BodyWeight+e,$$

and Batch (the date when the test is performed on mice) in the random effect term. We then apply the scoring method to obtain a consensus global inference from the multicentre results, following the logic described in the flowchart below (Fig. 3). We further compare our method with the global consensus criteria (all centres agree vs at least one centre disagree) and the random effects metadata analysis approach described in Cooper et al. (2009) (page 295–315) and (Stewart et al. 2012), implemented in the R package *metafor* (Viechtbauer 2010).

Table 4 shows the outcome of the scoring method for the 22 haematological parameters measured by the IMPC, as well as the comparison with a consensus method based on all centres agreeing on a significant sex effect and the meta-analysis method. Using the method proposed here, there is consensus among 11 IMPC centres for 14 traits with $-\mathrm{log}\left(s\right)>0$, with males on average higher than females for 9 traits (red blood cell count, red blood cell distribution width, haematocrit, platelet count, white blood cell count, lymphocyte cell count, neutrophil cell count, monocyte cell count, eosinophil cell count) and females on average higher than males for 5 traits (mean cell volume, mean corpuscular haemoglobin, mean cell haemoglobin concentration, mean platelet volume, and lymphocyte differential count). For 8 traits, the scoring method leads to zero or negative values, reflecting a lack of consensus (6 traits), or does not reach the minimum threshold of three centres providing measurements for the results to be processed (lack of information in the data—2 traits). The meta-analysis method shows consistent results with the scoring method, however, does not obtain the homogeneity of the statistical results across the centres for the monocyte cell count (also shown in Fig. 2 bottom), lymphocyte differential count and a borderline p-value for the eosinophil cell count (*p* value = 0.069) and the neutrophil differential count (*p* value = 0.048). Visual inspection of the data shows that the meta-analysis has a better performance for identifying the lack of agreement in *lymphocyte differential count* whereas the scoring method outperforms this method for the *monocyte cell count*. In contrast with the two methods above, the global consensus method shows the agreement across centres for the n*eutrophil cell count and Large Unstained Cell (LUC) count where the latter does not reach the requirement of a minimum of 3 centres.*

**Table 4:** The outcome of applying the scoring method to 22 haematological measurements collected by 11 IMPC centres compared with outcomes by the individual centre (first three columns) and a method based on measuring the heterogeneity of the SD estimates across the centres using random effects metadata analysis (last column). The traits are shown in rows followed by the counts for the centre-based statistical test results, the mean effect size for the 11 centres, the consensus score and the inference, which is based on the -log(score) and the sign of the mean effect size. The scoring method identifies consensus in sexual dimorphism across centres for 14 traits (green and red rows), no agreement for 8 traits (blue rows) and 2 traits which do not meet the minimum requirements for the calculation of the score (yellow rows). Only in 2 cases, all centres agree (in bold)

Trait name	Count of outcomes across centres			Do all centres agree?	Consensus score						Meta-analysis
	Not significant	Male higher	Female higher		Mean effect size	Score	-log(score)	Inference			Heterogeneity of the SD estimations across the centres p-value
Platelet count	1	10	0	No	1.25	0.45	0.35		Males Higher		< 0.01
White blood cell count	1	9	0	No	1.17	0.08	1.12		Males Higher		< 0.01
Lymphocyte cell count	1	5	0	No	1.01	0.14	0.86		Males Higher		< 0.01
Neutrophil cell count	0	6	0	**Yes**	0.80	0.02	1.71		Males Higher		< 0.01
Monocyte cell count	0	5	1	No	0.62	0.01	2.28		Males Higher		0.131
Red blood cell count	2	9	0	No	0.55	0.51	0.30		Males Higher		< 0.01
Red blood cell distribution width	1	7	0	No	0.53	0.18	0.74		Males Higher		< 0.01
Haematocrit	4	6	1	No	0.38	0.69	0.16		Males Higher		< 0.01
Eosinophil cell count	0	5	1	No	0.35	0.08	1.08		Males Higher		0.069
Lymphocyte differential count	2	1	3	No	-0.32	0.74	0.13		Female Higher		0.138
Mean cell volume	1	0	10	No	-0.47	0.38	0.42		Female Higher		< 0.01
Mean platelet volume	1	0	7	No	-0.51	0.60	0.22		Female Higher		< 0.01
Mean cell haemoglobin concentration	3	0	8	No	-0.52	0.73	0.14		Female Higher		< 0.01
Mean corpuscular haemoglobin	1	0	10	No	-0.90	0.23	0.64		Female Higher		< 0.01
Large Unstained Cell (LUC) count	0	3	0	**Yes**	-	-	-		Does not reach the minimum requirements for this analysis		< 0.01
Large Unstained Cell (LUC) differential count	2	1	0	No	-	-	-		Does not reach the minimum requirements for this analysis		0.111
Neutrophil differential count	3	2	1	No	0.35	1.16	-0.07		Not enough signal between or across centres to detect SD		0.048
Basophil cell count	1	3	1	No	0.25	1.00	0.00		Not enough signal between or across centres to detect SD		0.333
Haemoglobin	5	4	2	No	0.13	1.00	0.00		Not enough signal between or across centres to detect SD		0.147
Monocyte differential count	4	1	1	No	0.03	1.00	0.00		Not enough signal between or across centres to detect SD		0.709
Eosinophil differential count	4	1	1	No	-0.06	1.00	0.00		Not enough signal between or across centres to detect SD		0.603
Basophil differential count	2	1	2	No	-0.16	1.00	0.00		Not enough signal between or across centres to detect SD		0.220

### Conclusion and future work

Collecting data from multiple resources such as, in the case of this study, mouse phenotyping centres, benefits from a higher signal-to-noise ratio and a broader representation of a population. However, extra attention is required in the design and implementation of the experiments and statistical analysis to be able to make a global consensus inference from the aggregated results from individual resources (Rashid et al. 2012; Karp et al. 2017; Haselimashhadi et al. 2020a; Chung et al. 2010; Hu et al. 2022; Knatterud et al. 1998; Chalmers and Clarke 2004; Hogg 1991; Basagaña et al. 2018; Burke et al. 2017; Bowden et al. 2011; Stewart et al. 2012; Viechtbauer 2010; Bierer et al. 2017; Devereaux et al. 2016). Due to unavoidable, uncontrolled and unobserved factors, the results from all resources may only partially agree and a metric of consensus is required. In this paper, we propose a novel method which combines several aspects of multicentre experiment results including the corrected p-values, the magnitude and direction of effect sizes and the number of centres into one global consensus score.

We applied this method to identify sexual dimorphism in 22 haematological measurements collected from wildtype mice in 11 globally distributed centres forming part of the International Mouse Phenotyping Consortium (IMPC). We compared the results of this method to those obtained by the meta-analysis as well as by applying a binary method based on the agreement of all centres on the detection of sexual dimorphism. While the binary method found 2 traits reaching consensus across all IMPC centres, the method presented here allows to conclude sexual dimorphism in 14 traits, with males on average higher than females for 9 traits and females on average higher than males for 5 traits. Further, comparing our method with the meta-analysis method shows a high degree of overlap between the two $(\frac{16}{20}=80\%)$ for the haematological traits. Our method shows better performance for monocyte cell count ($-\mathrm{log}(\mathrm{score})=2.28$ versus meta-analysis *p*-value $= 0.131$) and eosinophil cell count ($-\mathrm{log}(\mathrm{score})=1.08$ versus meta-analysis *p*-value $=0.069$). However, a challenging case for the interpretation of the results is presented in comparing the outcome of the scoring method versus the meta-analysis method for lymphocyte differential count ($-\mathrm{log}(\mathrm{score})=0.13$ versus meta-analysis p-value $=0.138$). This study has focused on the IMPC haematology traits, but we believe the approach could be applied more generally and would be suitable to assess other IMPC parameters in the future.

### Future studies

In this study, we showed the application of our scoring method to IMPC haematological data. In future studies, we will investigate the performance of the method when applied to other IMPC procedures as well as obtaining the statistical properties of the test statistic. This will allow assigning a probability of consensus to the scores (in particular when they are close to 1 or -log(score) is close to zero) that contributes to the reliability of the method.

## Supplementary Information

Below is the link to the electronic supplementary material.

Supplementary file1 (PDF 2516 kb)

## Data Availability

All data used in the study correspond to the IMPC data release 15.1 (October 2021) and can be retrieved from the IMPC data repository under the URL https://www.mousephenotype.org/help/non-programmatic-data-access/. A copy of the data, results and source codes are publicly available from www.doi.org/10.5281/zenodo.7704684.
