# Supplementary material for: A consensus score to combine inferences from multiple centres
Source: Mamm Genome. 2023 May 8;34(3):379–88. doi: 10.1007/s00335-023-09993-0 (PMC10382396; doi:10.1007/s00335-023-09993-0)
Supplement: Supplementary file 1 — Supplementary file1 (PDF 2516 kb) [file 335_2023_9993_MOESM1_ESM.pdf]

Supplementary Figure 1. The data variation for the IMPC wildtype mice over time (batch) for the red blood cell counts across the IMPC centres. The global mean is shown by the vertical blue lines. The IMPC centres show some variations over time. Align with [1] a batch effect term is included in the analysis to account for the day to day variation in the data.

1. Karp NA, Mason J, Beaudet AL, Benjamini Y, Bower L, Braun RE, et al. Prevalence of sexual dimorphism in mammalian phenotypic traits. Nat Commun. 2017;8: 15475. doi:10.1038/ncomms15475

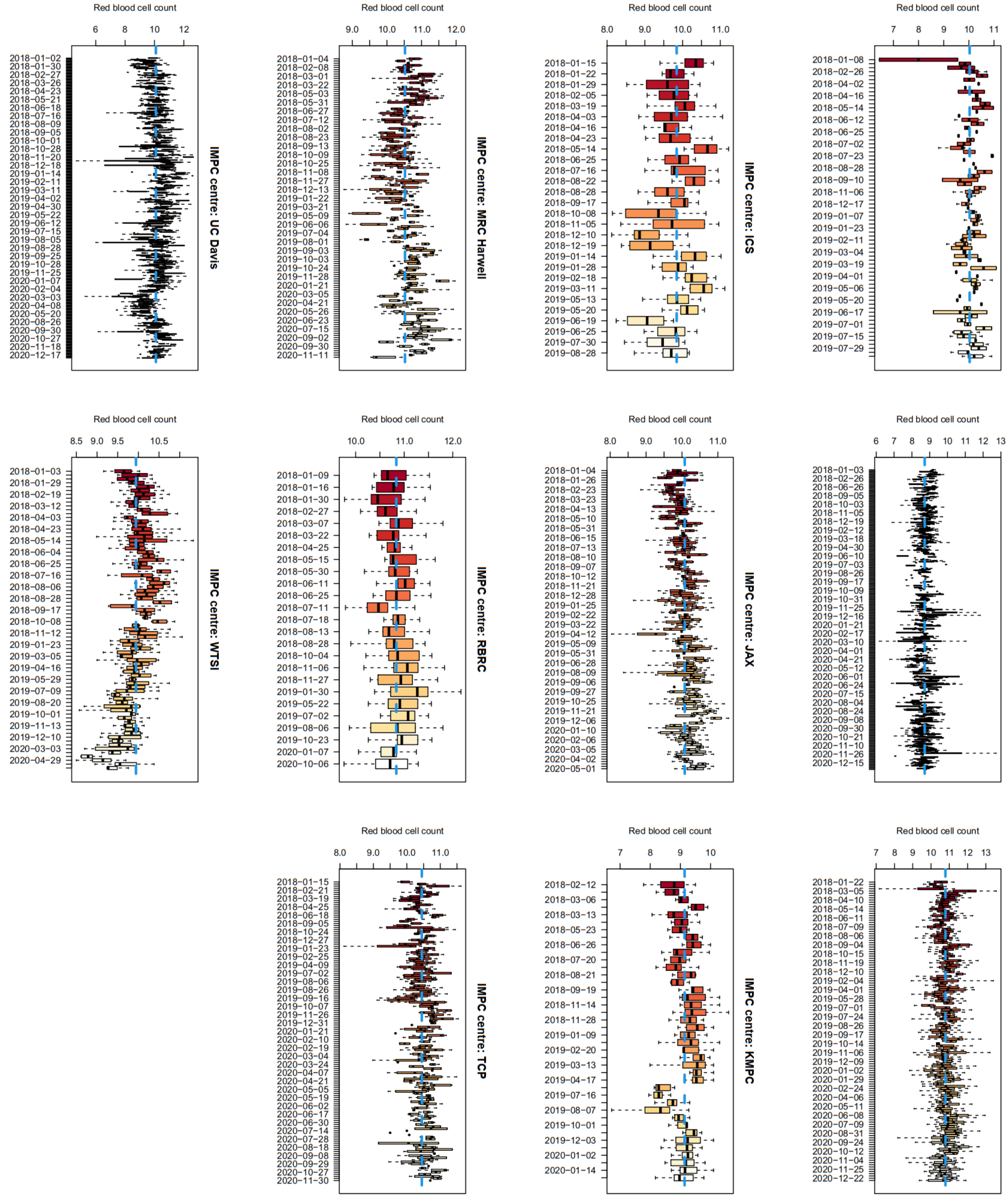

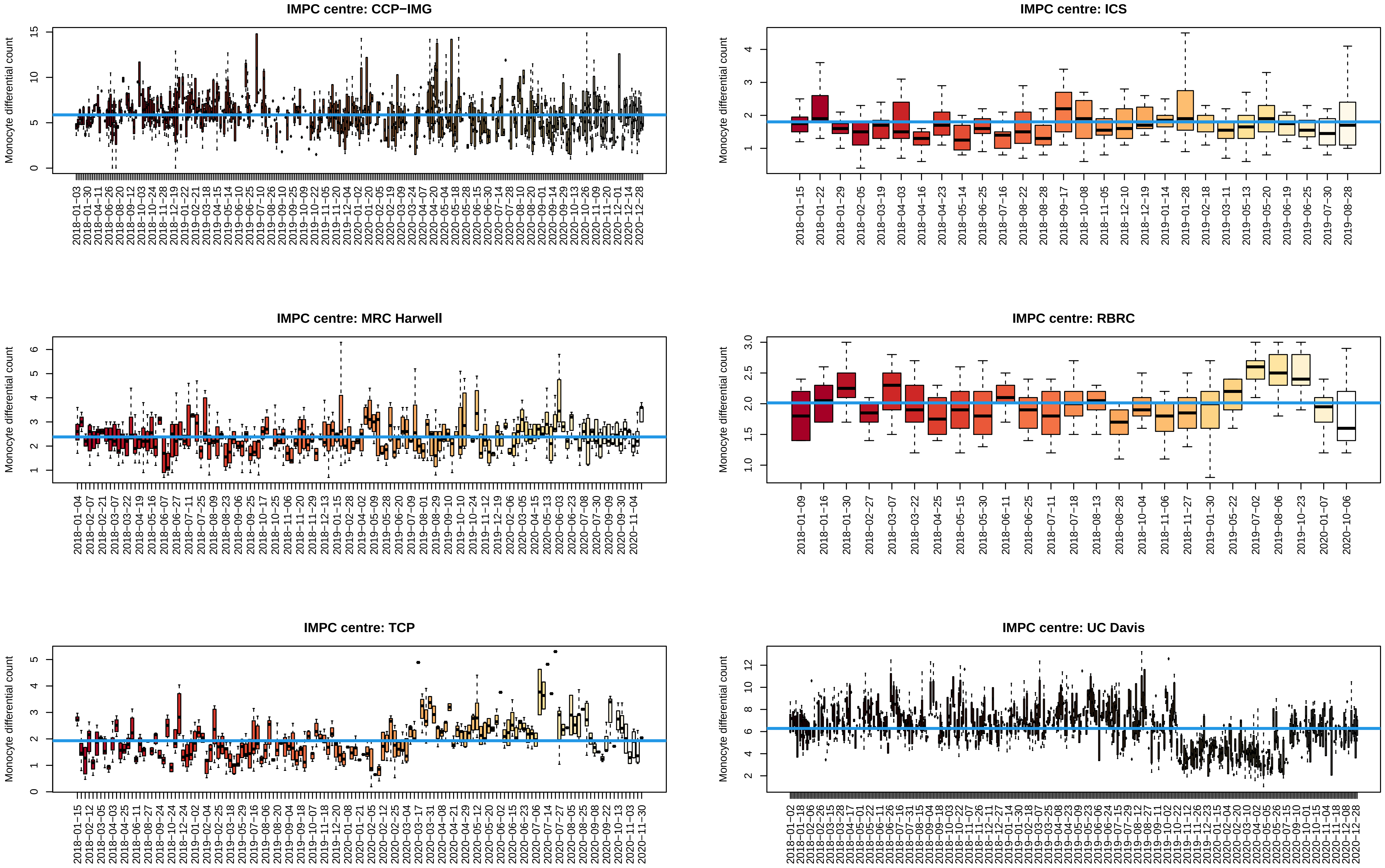
IMPC centre: CCP−IMG
Monocyte differential count
IMPC centre: ICS
Monocyte differential count
2018−01−15
2018−01−22
2018−01−29
2018−02−05
2018−03−19
2018−04−03
2018−04−16
2018−04−23
2018−05−14
2018−06−25
2018−07−16
2018−08−22
2018−08−28
2018−09−17
2018−10−08
2018−11−05
2018−12−10
2018−12−19
2019−01−14
2019−01−28
2019−02−18
2019−03−11
2019−05−13
2019−05−20
2019−06−19
2019−06−25
2019−07−30
2019−08−28
IMPC centre: MRC Harwell
Monocyte differential count
IMPC centre: RBRC
Monocyte differential count
2018−01−09
2018−01−16
2018−01−30
2018−02−27
2018−03−07
2018−03−22
2018−04−25
2018−05−15
2018−05−30
2018−06−11
2018−06−25
2018−07−11
2018−07−18
2018−08−13
2018−08−28
2018−10−04
2018−11−06
2018−11−27
2019−01−30
2019−05−22
2019−07−02
2019−08−06
2019−10−23
2020−01−07
2020−10−06
IMPC centre: TCP
Monocyte differential count
IMPC centre: UC Davis
Monocyte differential count

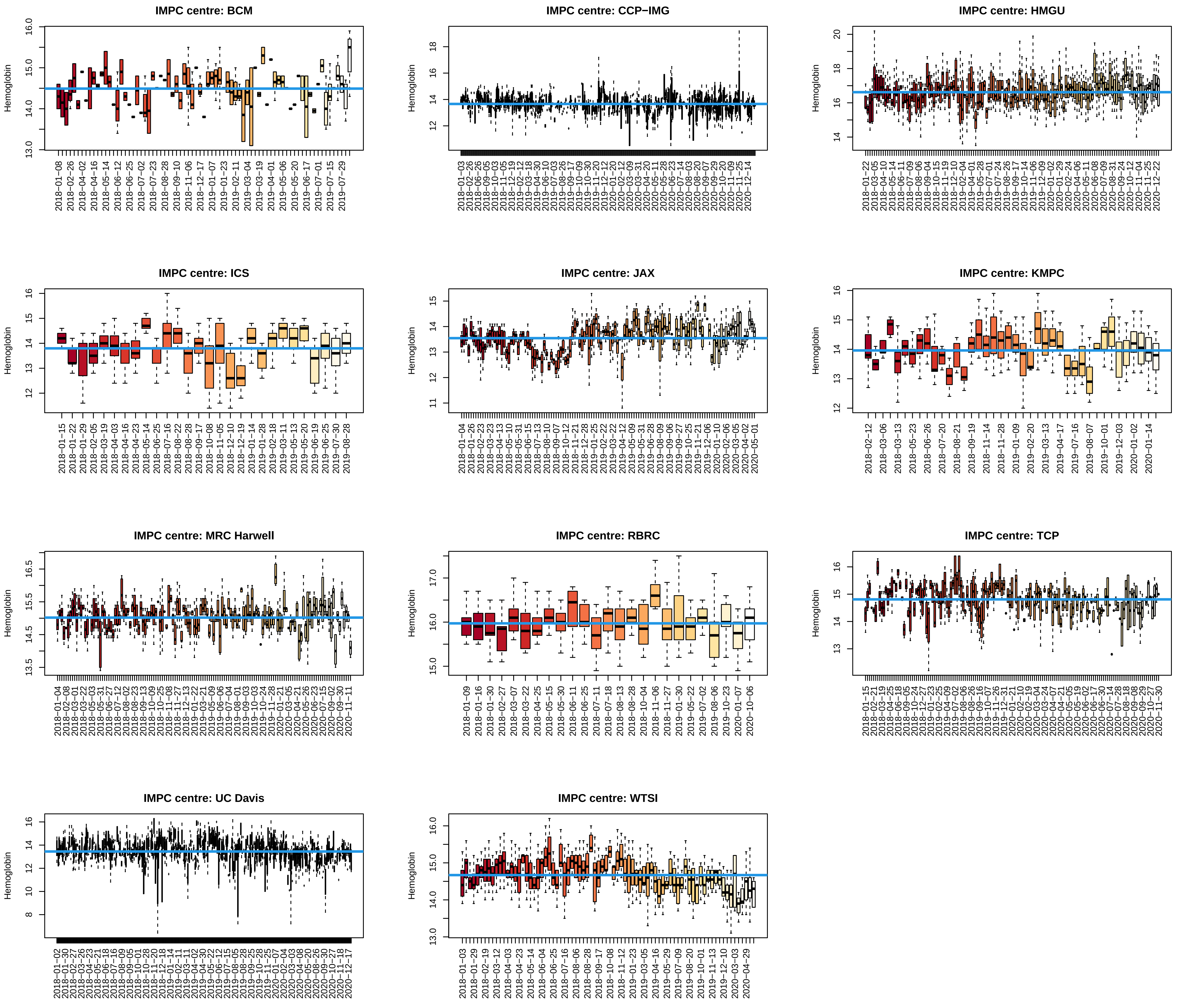
IMPC centre: BCM
IMPC centre: CCP−IMG
IMPC centre: HMGU
IMPC centre: ICS
IMPC centre: JAX
IMPC centre: KMPC
IMPC centre: MRC Harwell
IMPC centre: RBRC
IMPC centre: TCP
IMPC centre: UC Davis
IMPC centre: WTSI
Hemoglobin

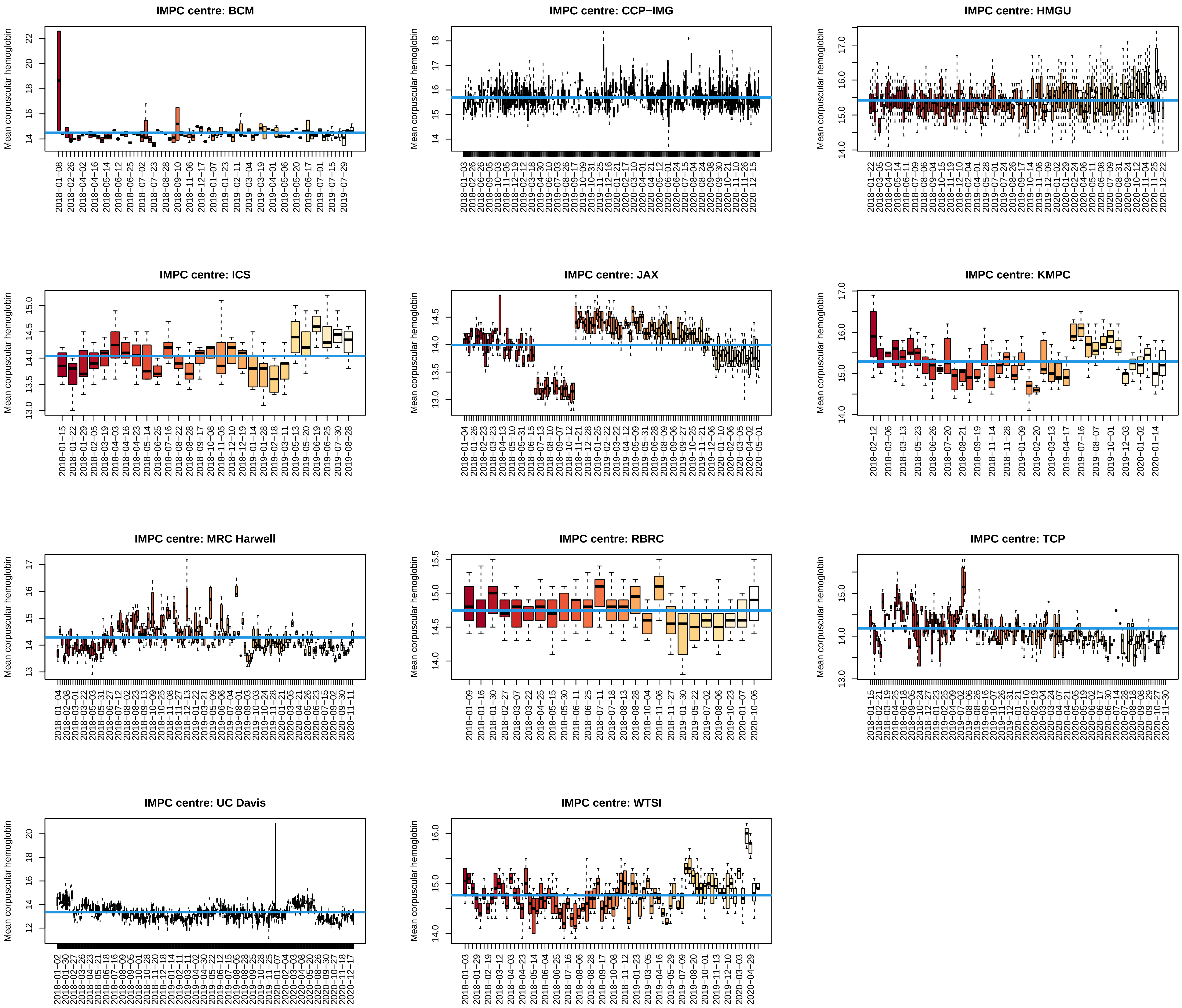
IMPC centre: BCM
IMPC centre: CCP−IMG
IMPC centre: HMGU
IMPC centre: ICS
IMPC centre: JAX
IMPC centre: KMPC
IMPC centre: MRC Harwell
IMPC centre: RBRC
IMPC centre: TCP
IMPC centre: UC Davis
IMPC centre: WTSI
Mean corpuscular hemoglobin

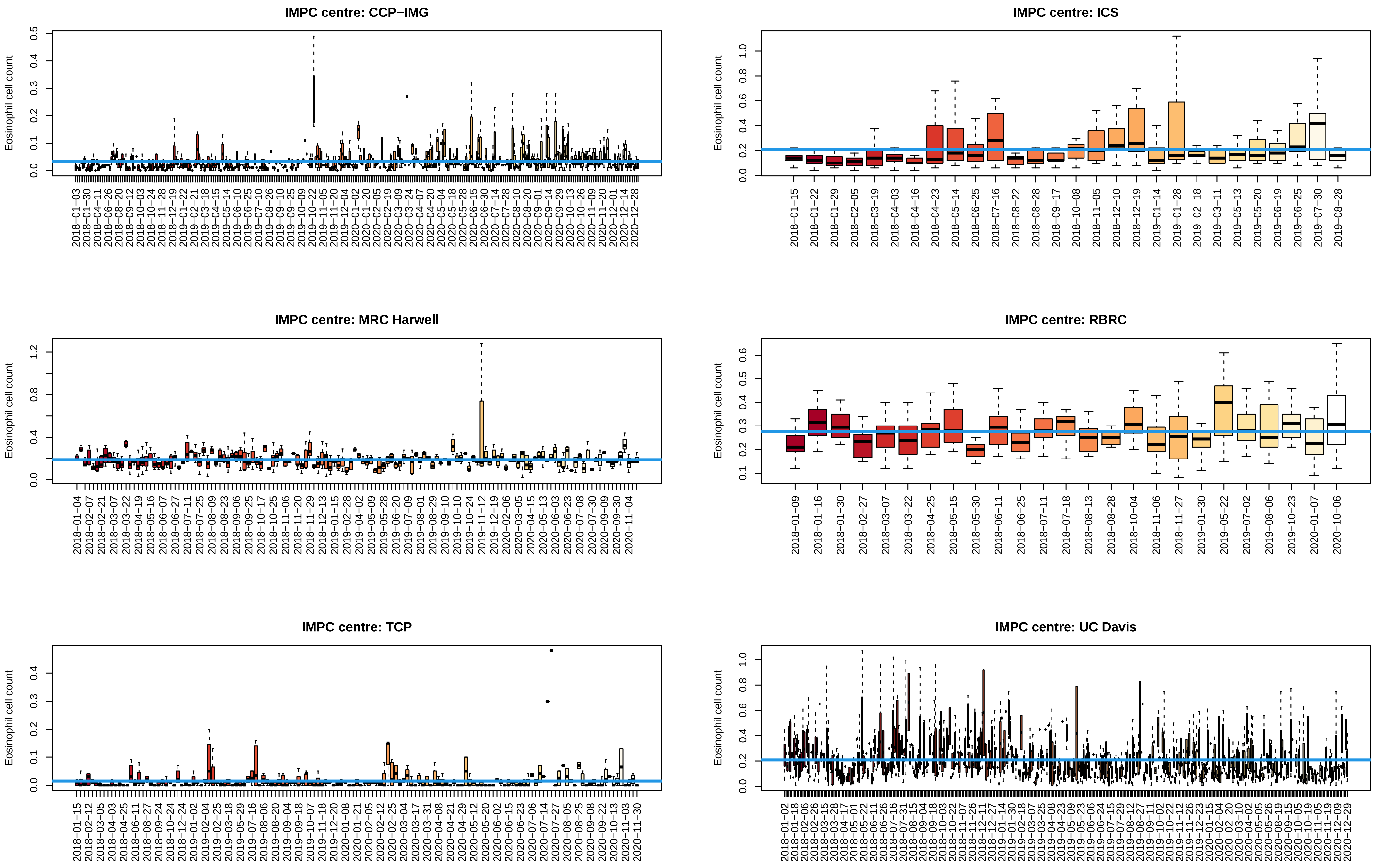
IMPC centre: CCP−IMG
Eosinophil cell count
IMPC centre: ICS
Eosinophil cell count
IMPC centre: MRC Harwell
Eosinophil cell count
IMPC centre: RBRC
Eosinophil cell count
IMPC centre: TCP
Eosinophil cell count
IMPC centre: UC Davis
Eosinophil cell count

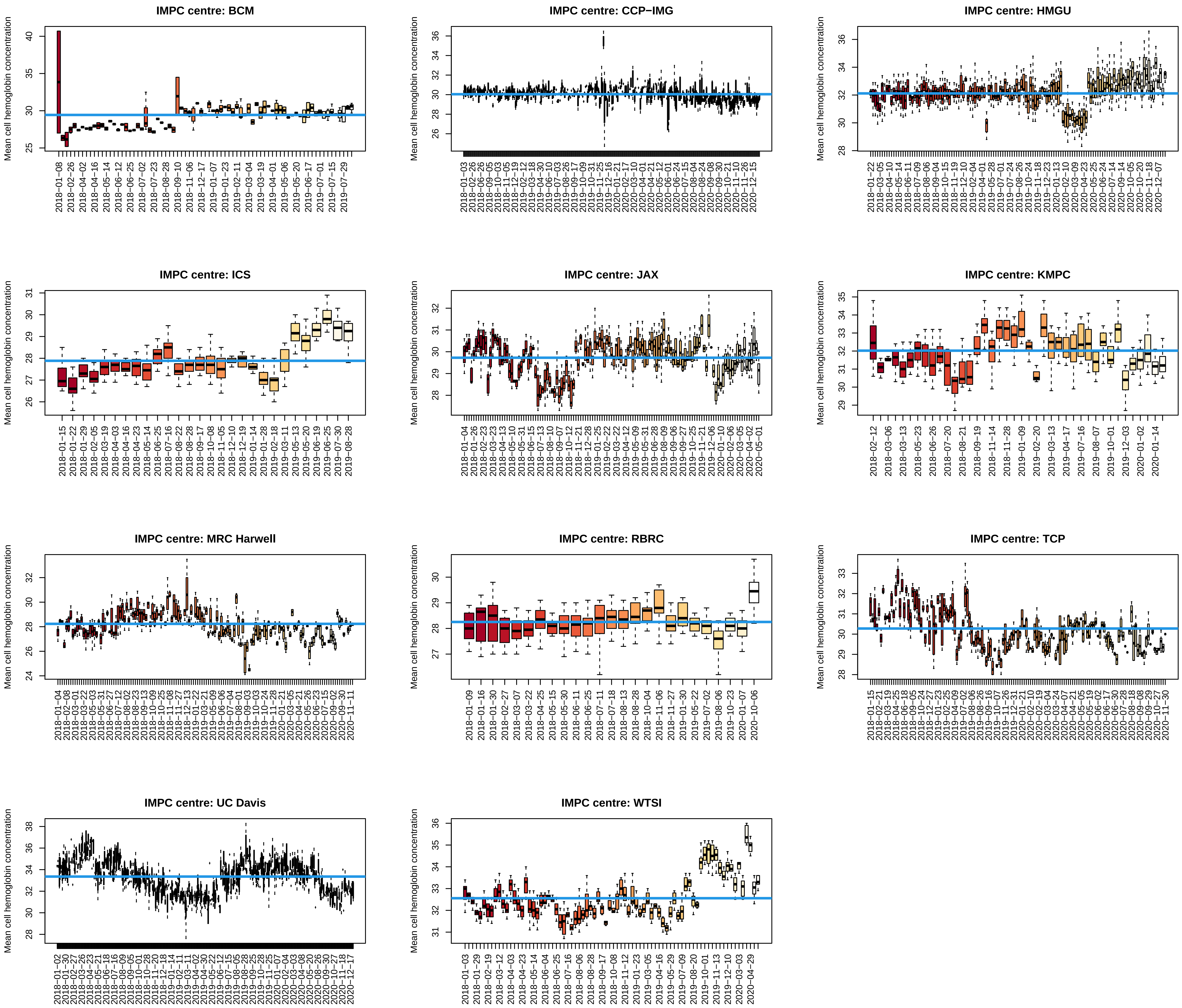
IMPC centre: BCM
IMPC centre: CCP−IMG
IMPC centre: HMGU
IMPC centre: ICS
IMPC centre: JAX
IMPC centre: KMPC
IMPC centre: MRC Harwell
IMPC centre: RBRC
IMPC centre: TCP
IMPC centre: UC Davis
IMPC centre: WTSI
Mean cell hemoglobin concentration

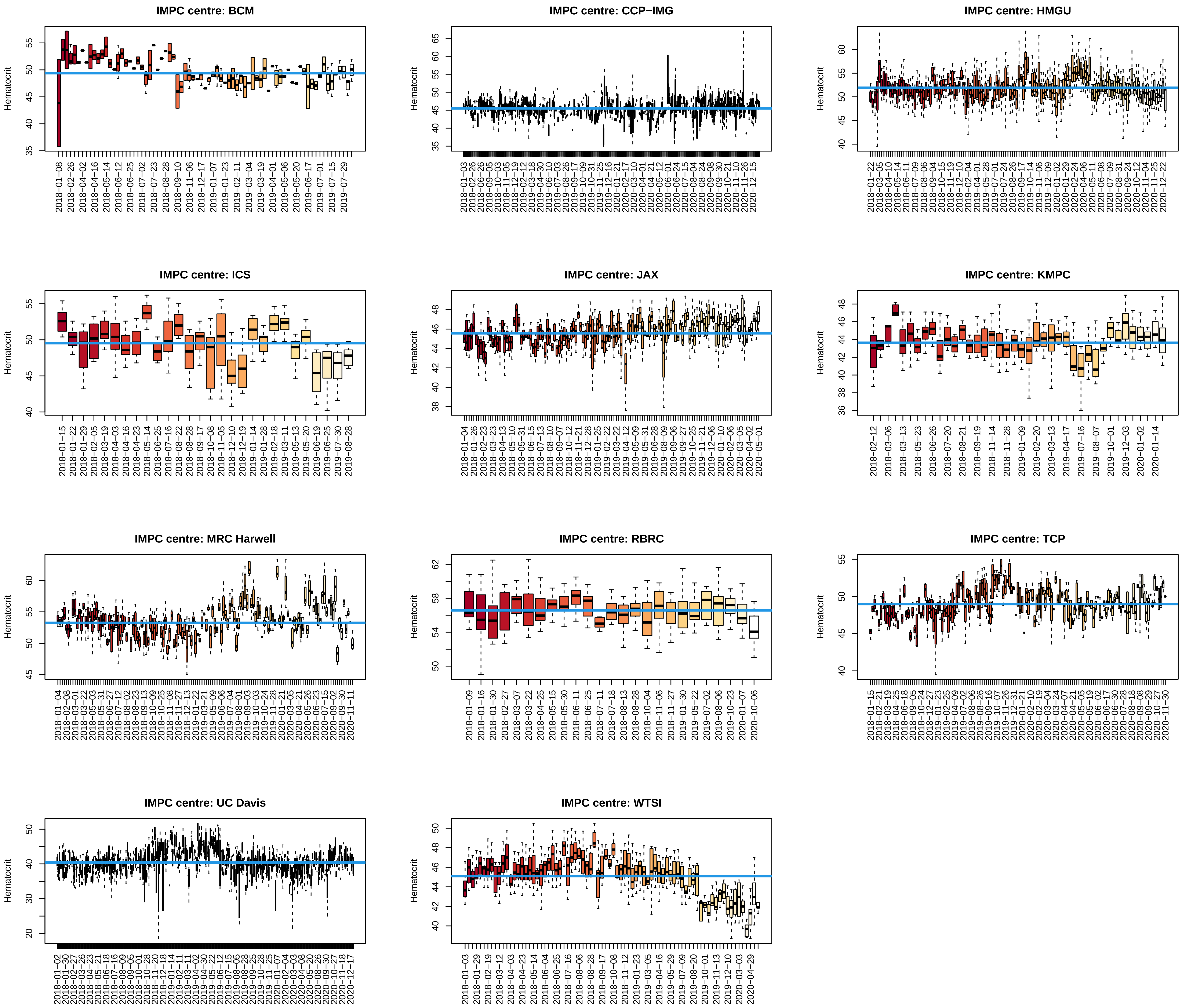
IMPC centre: BCM
IMPC centre: CCP−IMG
IMPC centre: HMGU
IMPC centre: ICS
IMPC centre: JAX
IMPC centre: KMPC
IMPC centre: MRC Harwell
IMPC centre: RBRC
IMPC centre: TCP
IMPC centre: UC Davis
IMPC centre: WTSI
Hematocrit

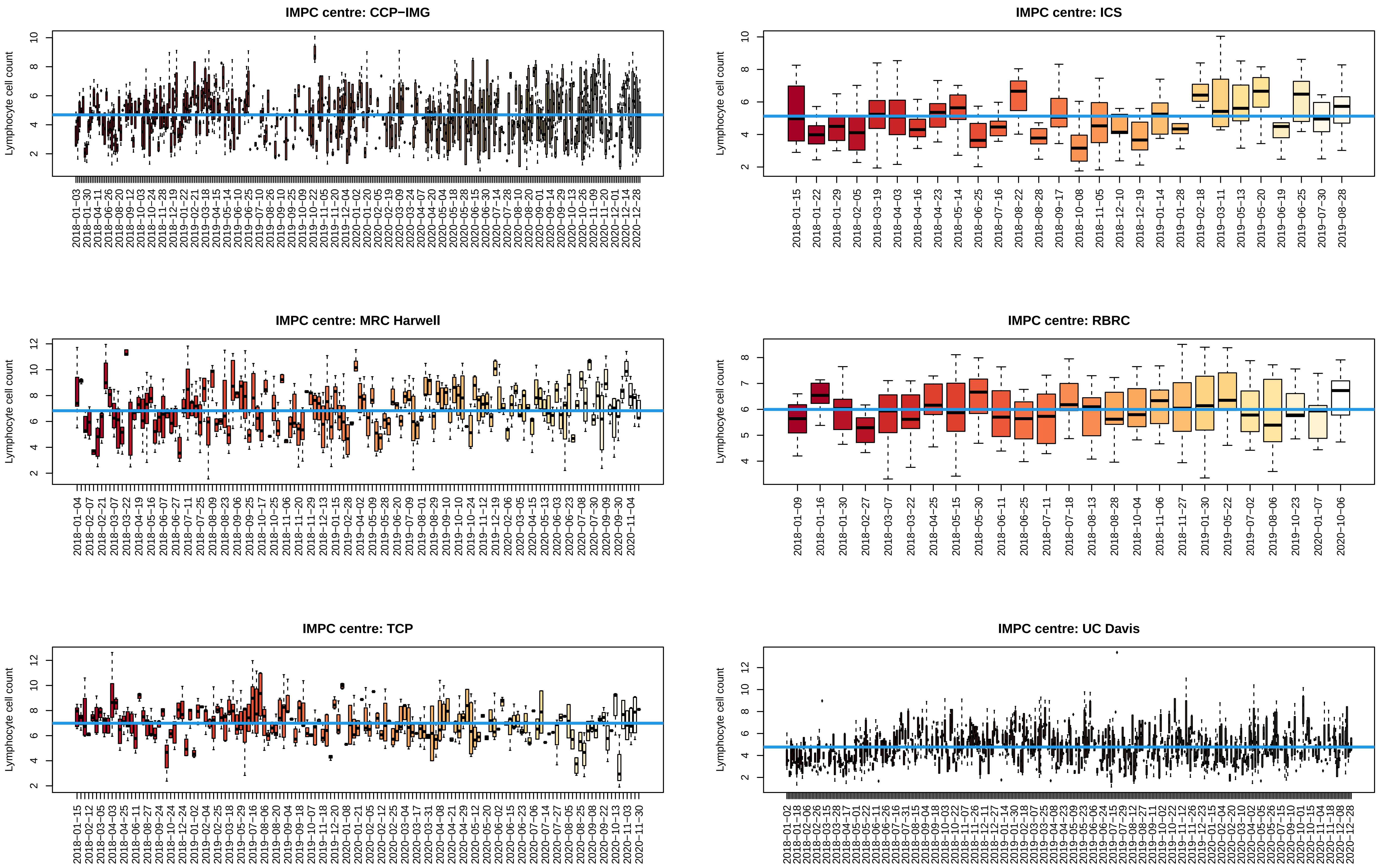
IMPC centre: CCP−IMG
Lymphocyte cell count
IMPC centre: ICS
Lymphocyte cell count
IMPC centre: MRC Harwell
Lymphocyte cell count
IMPC centre: RBRC
Lymphocyte cell count
IMPC centre: TCP
Lymphocyte cell count
IMPC centre: UC Davis
Lymphocyte cell count

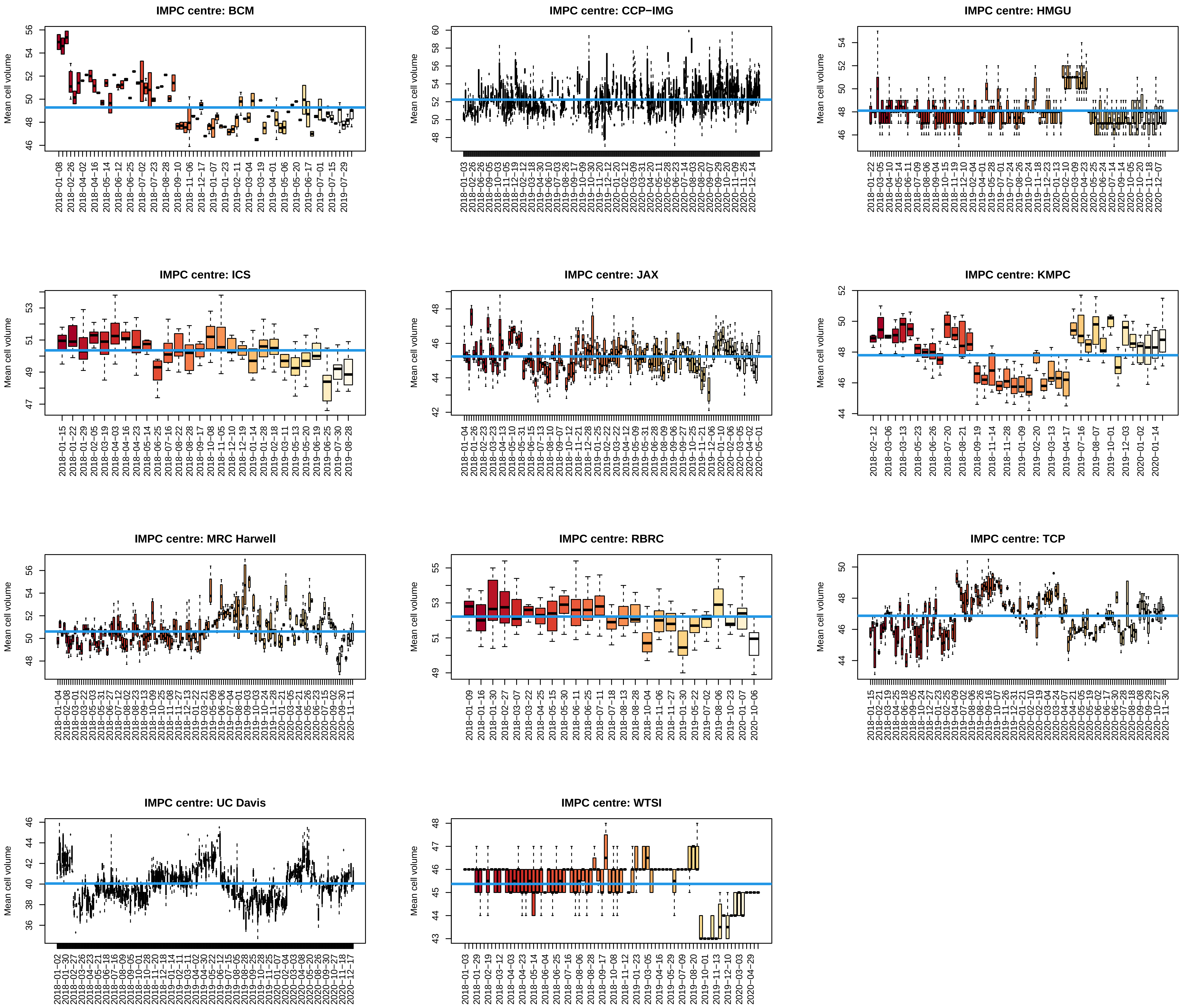
IMPC centre: BCM
IMPC centre: CCP−IMG
IMPC centre: HMGU
IMPC centre: ICS
IMPC centre: JAX
IMPC centre: KMPC
IMPC centre: MRC Harwell
IMPC centre: RBRC
IMPC centre: TCP
IMPC centre: UC Davis
IMPC centre: WTSI
Mean cell volume

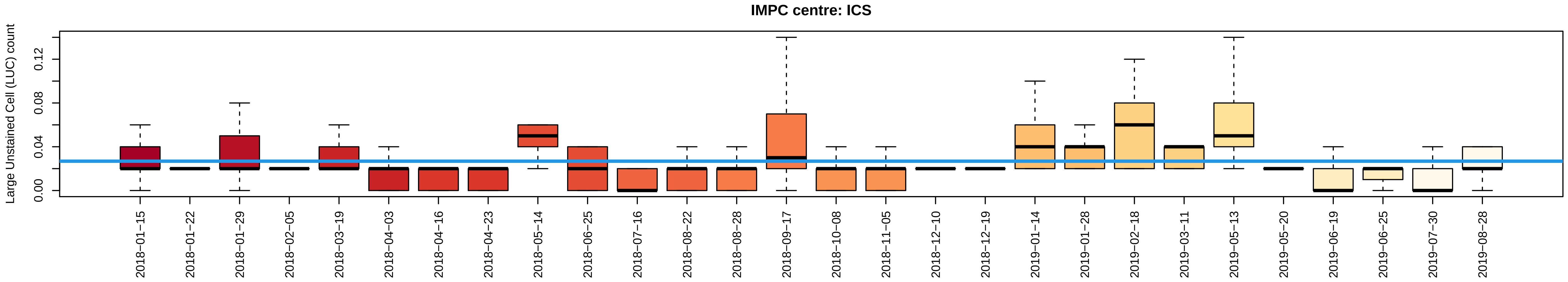
IMPC centre: ICS
Large Unstained Cell (LUC) count
0.00
0.04
0.08
0.12
2018-01-15
2018-01-22
2018-01-29
2018-02-05
2018-03-19
2018-04-03
2018-04-16
2018-04-23
2018-05-14
2018-06-25
2018-07-16
2018-08-22
2018-08-28
2018-09-17
2018-10-08
2018-11-05
2018-12-10
2018-12-19
2019-01-14
2019-01-28
2019-02-18
2019-03-11
2019-05-13
2019-05-20
2019-06-19
2019-06-25
2019-07-30
2019-08-28

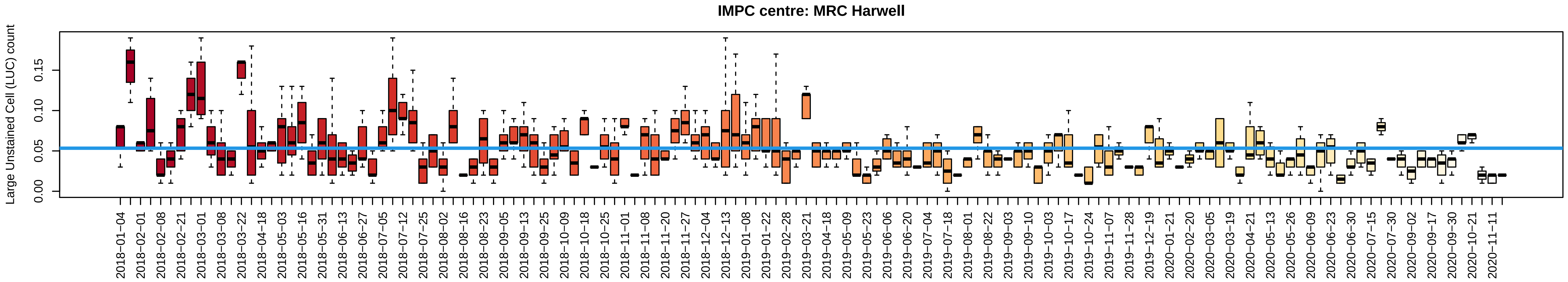
IMPC centre: MRC Harwell
Large Unstained Cell (LUC) count
0.00
0.05
0.10
0.15
2018-01-04
2018-02-01
2018-02-08
2018-02-21
2018-03-01
2018-03-08
2018-03-22
2018-04-18
2018-05-03
2018-05-16
2018-05-31
2018-06-13
2018-06-27
2018-07-05
2018-07-12
2018-07-25
2018-08-02
2018-08-16
2018-08-23
2018-09-05
2018-09-13
2018-09-25
2018-10-09
2018-10-18
2018-10-25
2018-11-01
2018-11-08
2018-11-20
2018-11-27
2018-12-04
2018-12-13
2019-01-08
2019-01-22
2019-02-28
2019-03-21
2019-04-18
2019-05-09
2019-05-23
2019-06-06
2019-06-20
2019-07-04
2019-07-18
2019-08-01
2019-08-22
2019-09-03
2019-09-10
2019-10-03
2019-10-17
2019-10-24
2019-11-07
2019-11-28
2019-12-19
2020-01-21
2020-02-20
2020-03-05
2020-03-19
2020-04-21
2020-05-13
2020-05-26
2020-06-09
2020-06-23
2020-06-30
2020-07-15
2020-07-30
2020-09-02
2020-09-17
2020-09-30
2020-10-21
2020-11-11

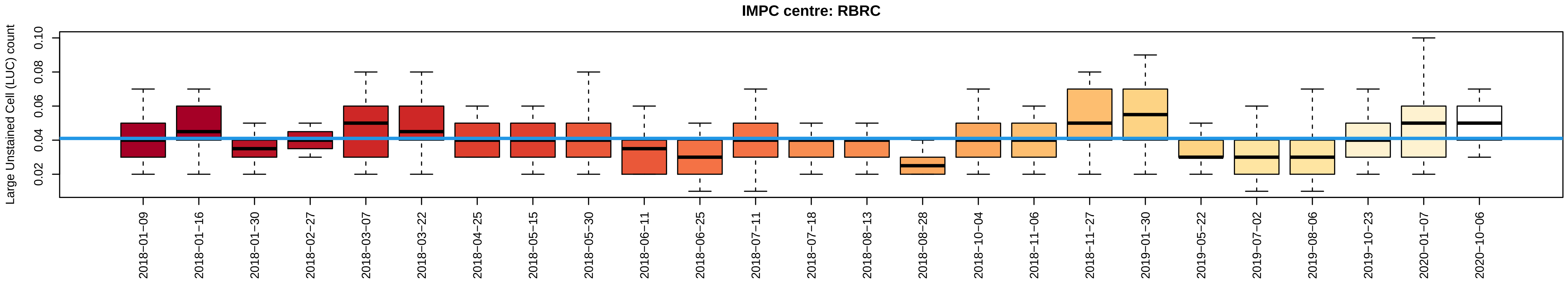
IMPC centre: RBRC
Large Unstained Cell (LUC) count
0.02
0.04
0.06
0.08
0.10
2018-01-09
2018-01-16
2018-01-30
2018-02-27
2018-03-07
2018-03-22
2018-04-25
2018-05-15
2018-05-30
2018-06-11
2018-06-25
2018-07-11
2018-07-18
2018-08-13
2018-08-28
2018-10-04
2018-11-06
2018-11-27
2019-01-30
2019-05-22
2019-07-02
2019-08-06
2019-10-23
2020-01-07
2020-10-06

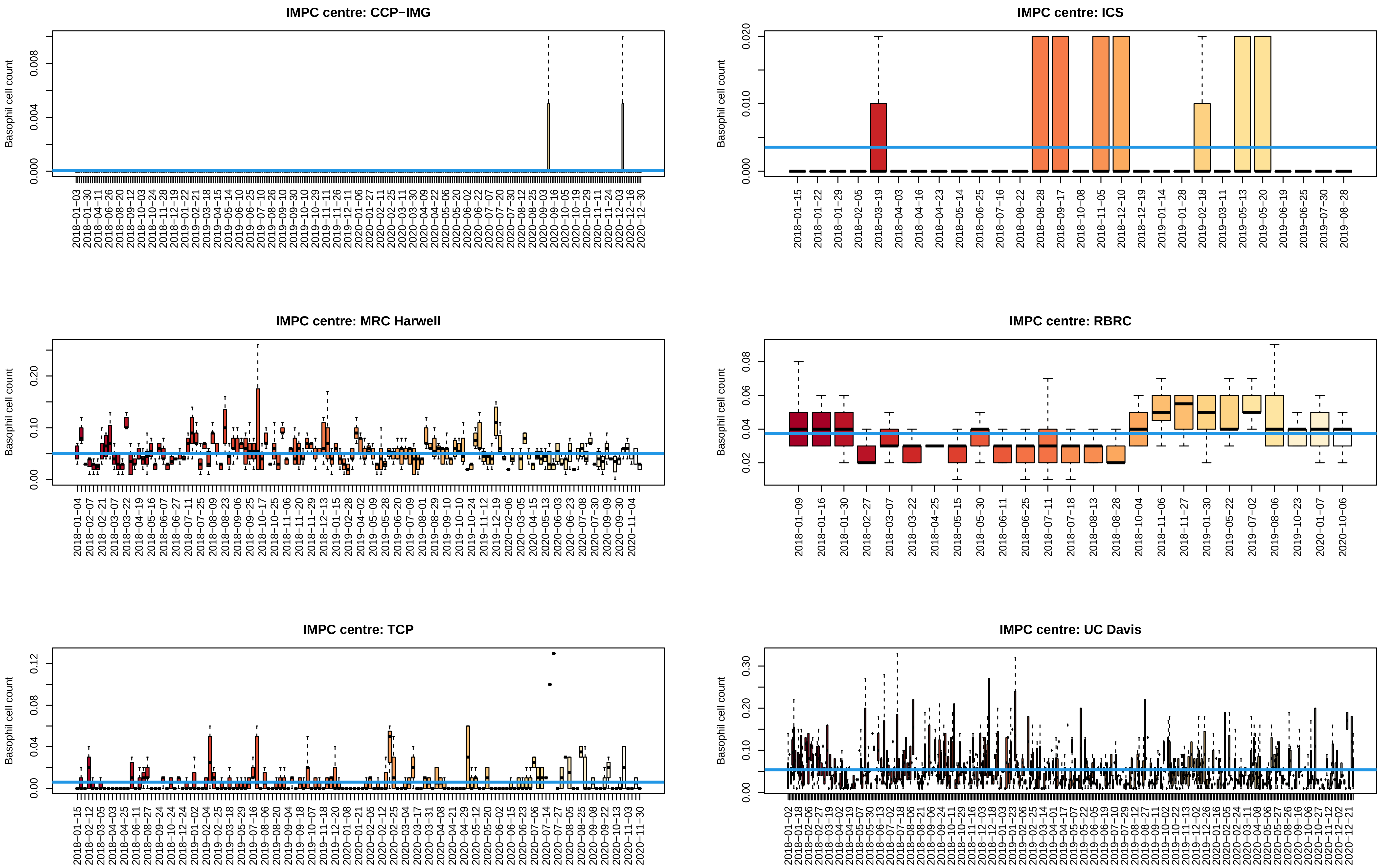

IMPC centre: CCP−IMG
Basophil cell count
0.000
0.004
0.008
2018−01−03 2018−01−30 2018−04−11 2018−06−26 2018−08−20 2018−09−12 2018−10−03 2018−10−24 2018−11−28 2018−12−19 2019−01−22 2019−02−21 2019−03−18 2019−04−15 2019−05−14 2019−06−10 2019−06−25 2019−07−10 2019−08−26 2019−09−10 2019−09−30 2019−10−10 2019−10−29 2019−11−11 2019−11−26 2019−12−11 2020−01−06 2020−01−27 2020−02−11 2020−02−25 2020−03−11 2020−03−30 2020−04−09 2020−04−22 2020−05−06 2020−05−20 2020−06−02 2020−06−22 2020−07−07 2020−07−20 2020−07−30 2020−08−12 2020−08−25 2020−09−03 2020−09−16 2020−10−05 2020−10−19 2020−10−29 2020−11−11 2020−11−24 2020−12−03 2020−12−16 2020−12−30
IMPC centre: ICS
Basophil cell count
0.000
0.010
0.020
2018−01−15 2018−01−22 2018−01−29 2018−02−05 2018−03−19 2018−04−03 2018−04−16 2018−04−23 2018−05−14 2018−06−25 2018−07−16 2018−08−22 2018−08−28 2018−09−17 2018−10−08 2018−11−05 2018−12−10 2018−12−19 2019−01−14 2019−01−28 2019−02−18 2019−03−11 2019−05−13 2019−05−20 2019−06−19 2019−06−25 2019−07−30 2019−08−28
IMPC centre: MRC Harwell
Basophil cell count
0.00
0.10
0.20
2018−01−04 2018−02−07 2018−02−21 2018−03−07 2018−03−22 2018−04−19 2018−05−16 2018−06−07 2018−06−27 2018−07−11 2018−07−25 2018−08−09 2018−08−23 2018−09−06 2018−09−25 2018−10−17 2018−10−25 2018−11−06 2018−11−20 2018−11−29 2018−12−13 2019−01−15 2019−02−28 2019−04−02 2019−05−09 2019−05−28 2019−06−20 2019−07−09 2019−08−01 2019−08−29 2019−09−10 2019−10−10 2019−10−24 2019−11−12 2019−12−19 2020−02−06 2020−03−05 2020−04−15 2020−05−13 2020−06−03 2020−06−23 2020−07−08 2020−07−30 2020−09−09 2020−09−30 2020−11−04
IMPC centre: RBRC
Basophil cell count
0.02
0.04
0.06
0.08
2018−01−09 2018−01−16 2018−01−30 2018−02−27 2018−03−07 2018−03−22 2018−04−25 2018−05−15 2018−05−30 2018−06−11 2018−06−25 2018−07−11 2018−07−18 2018−08−13 2018−08−28 2018−10−04 2018−11−06 2018−11−27 2019−01−30 2019−05−22 2019−07−02 2019−08−06 2019−10−23 2020−01−07 2020−10−06
IMPC centre: TCP
Basophil cell count
0.00
0.04
0.08
0.12
2018−01−15 2018−02−12 2018−03−05 2018−04−03 2018−04−25 2018−06−11 2018−08−27 2018−09−24 2018−10−24 2018−12−24 2019−01−02 2019−02−04 2019−02−25 2019−03−18 2019−05−29 2019−07−16 2019−08−06 2019−08−20 2019−09−04 2019−09−18 2019−10−07 2019−11−18 2019−12−20 2020−01−08 2020−01−21 2020−02−05 2020−02−12 2020−02−25 2020−03−04 2020−03−17 2020−03−31 2020−04−08 2020−04−21 2020−04−29 2020−05−12 2020−05−20 2020−06−02 2020−06−15 2020−06−23 2020−07−06 2020−07−14 2020−07−27 2020−08−05 2020−08−25 2020−09−08 2020−09−22 2020−10−13 2020−11−03 2020−11−30
IMPC centre: UC Davis
Basophil cell count
0.00
0.10
0.20
0.30

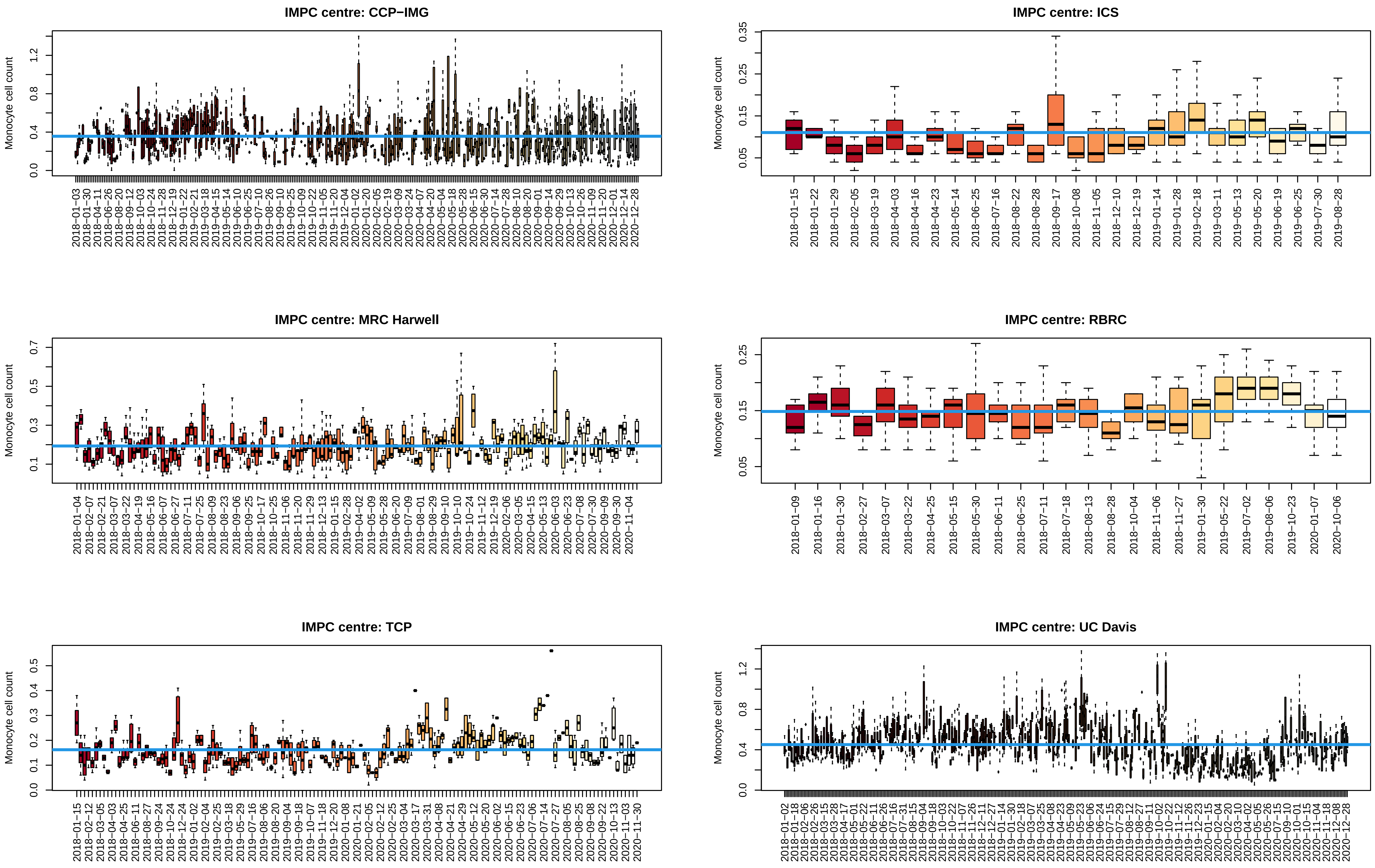
IMPC centre: CCP−IMG
IMPC centre: ICS
IMPC centre: MRC Harwell
IMPC centre: RBRC
IMPC centre: TCP
IMPC centre: UC Davis
Monocyte cell count
0.0
0.4
0.8
1.2
0.05
0.15
0.25
0.35
0.1
0.3
0.5
0.7
0.2

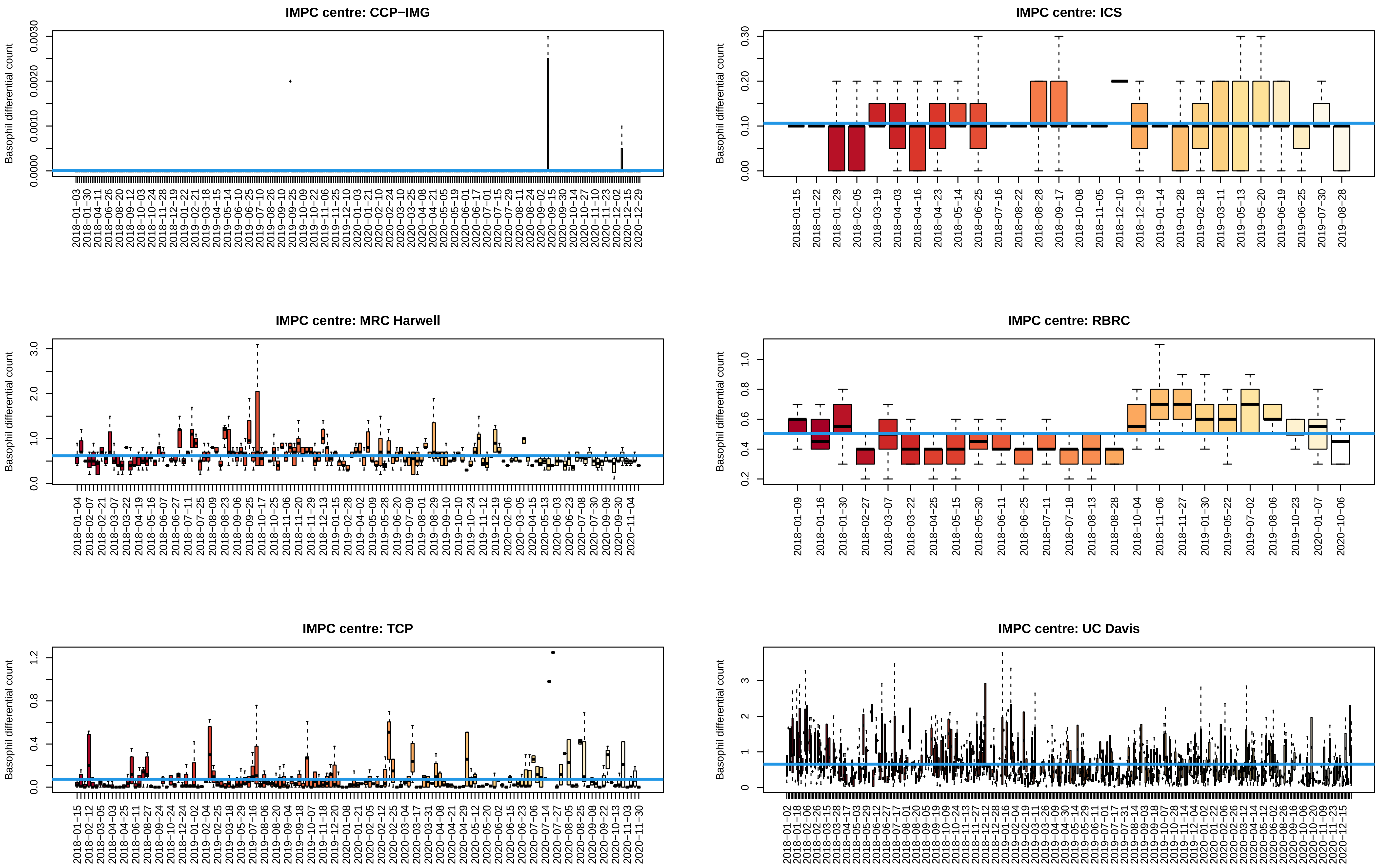
IMPC centre: CCP−IMG
Basophil differential count
0.0000 0.0010 0.0020 0.0030
2018−01−03 2018−01−30 2018−04−11 2018−06−26 2018−08−20 2018−09−12 2018−10−03 2018−10−24 2018−11−28 2018−12−19 2019−01−22 2019−02−21 2019−03−18 2019−04−15 2019−05−14 2019−06−10 2019−06−25 2019−07−10 2019−08−26 2019−09−10 2019−09−25 2019−10−09 2019−10−22 2019−11−06 2019−11−25 2019−12−10 2020−01−03 2020−01−21 2020−02−10 2020−02−24 2020−03−10 2020−03−25 2020−04−08 2020−04−21 2020−05−05 2020−05−19 2020−06−01 2020−06−17 2020−07−01 2020−07−15 2020−07−29 2020−08−11 2020−08−24 2020−09−02 2020−09−15 2020−09−30 2020−10−14 2020−10−27 2020−11−10 2020−11−23 2020−12−02 2020−12−15 2020−12−29
IMPC centre: ICS
Basophil differential count
0.00 0.10 0.20 0.30
2018−01−15 2018−01−22 2018−01−29 2018−02−05 2018−03−19 2018−04−03 2018−04−16 2018−04−23 2018−05−14 2018−06−25 2018−07−16 2018−08−22 2018−08−28 2018−09−17 2018−10−08 2018−11−05 2018−12−10 2018−12−19 2019−01−14 2019−01−28 2019−02−18 2019−03−11 2019−05−13 2019−05−20 2019−06−19 2019−06−25 2019−07−30 2019−08−28
IMPC centre: MRC Harwell
Basophil differential count
0.0 1.0 2.0 3.0
2018−01−04 2018−02−07 2018−02−21 2018−03−07 2018−03−22 2018−04−19 2018−05−16 2018−06−07 2018−06−27 2018−07−11 2018−07−25 2018−08−09 2018−08−23 2018−09−06 2018−09−25 2018−10−17 2018−10−25 2018−11−06 2018−11−20 2018−11−29 2018−12−13 2019−01−15 2019−02−28 2019−04−02 2019−05−09 2019−05−28 2019−06−20 2019−07−09 2019−08−01 2019−08−29 2019−09−10 2019−10−10 2019−10−24 2019−11−12 2019−12−19 2020−02−06 2020−03−05 2020−04−15 2020−05−13 2020−06−03 2020−06−23 2020−07−08 2020−07−30 2020−09−09 2020−09−30 2020−11−04
IMPC centre: RBRC
Basophil differential count
0.2 0.4 0.6 0.8 1.0
2018−01−09 2018−01−16 2018−01−30 2018−02−27 2018−03−07 2018−03−22 2018−04−25 2018−05−15 2018−05−30 2018−06−11 2018−06−25 2018−07−11 2018−07−18 2018−08−13 2018−08−28 2018−10−04 2018−11−06 2018−11−27 2019−01−30 2019−05−22 2019−07−02 2019−08−06 2019−10−23 2020−01−07 2020−10−06
IMPC centre: TCP
Basophil differential count
0.0 0.4 0.8 1.2
2018−01−15 2018−02−12 2018−03−05 2018−04−03 2018−04−25 2018−06−11 2018−08−27 2018−09−24 2018−10−24 2018−12−24 2019−01−02 2019−02−04 2019−02−25 2019−03−18 2019−05−29 2019−07−16 2019−08−06 2019−08−20 2019−09−04 2019−09−18 2019−10−07 2019−11−18 2019−12−20 2020−01−08 2020−01−21 2020−02−05 2020−02−12 2020−02−25 2020−03−04 2020−03−17 2020−03−31 2020−04−08 2020−04−21 2020−04−29 2020−05−12 2020−05−20 2020−06−02 2020−06−15 2020−06−23 2020−07−06 2020−07−14 2020−07−27 2020−08−05 2020−08−25 2020−09−08 2020−09−22 2020−10−13 2020−11−03 2020−11−30
IMPC centre: UC Davis
Basophil differential count
0 1 2 3
2018−01−02 2018−01−18 2018−02−06 2018−02−26 2018−03−15 2018−03−28 2018−04−17 2018−05−03 2018−05−29 2018−06−12 2018−06−27 2018−07−17 2018−08−01 2018−08−20 2018−09−05 2018−09−19 2018−10−09 2018−10−24 2018−11−13 2018−11−27 2018−12−12 2018−12−28 2019−01−16 2019−02−04 2019−02−19 2019−03−11 2019−03−26 2019−04−09 2019−04−30 2019−05−14 2019−05−29 2019−06−11 2019−07−01 2019−07−17 2019−07−31 2019−08−14 2019−09−03 2019−09−18 2019−10−08 2019−10−28 2019−11−14 2019−12−04 2020−01−02 2020−01−22 2020−02−06 2020−02−26 2020−03−12 2020−04−14 2020−05−12 2020−06−02 2020−08−26 2020−09−16 2020−10−06 2020−10−20 2020−11−09 2020−11−23 2020−12−15

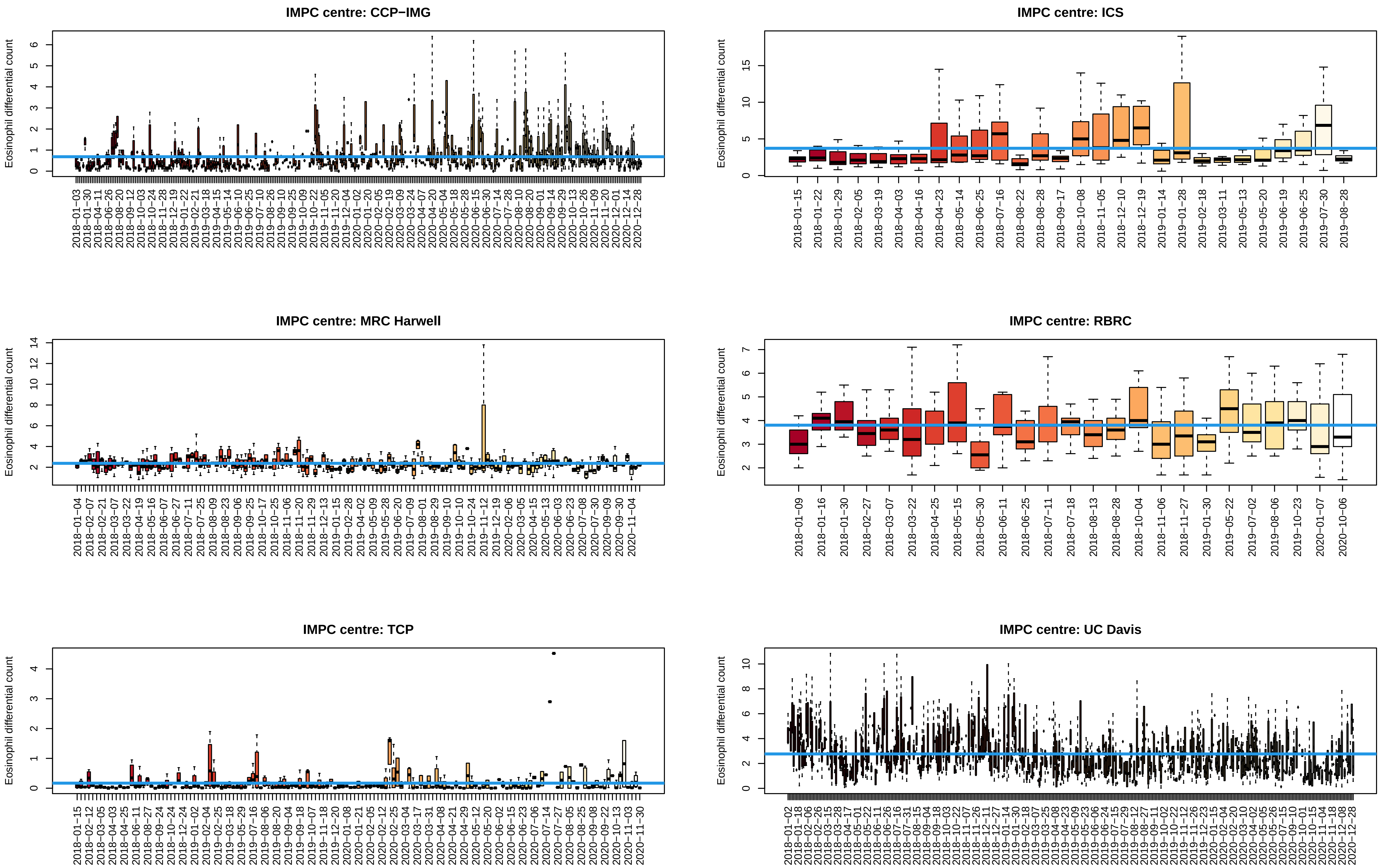
IMPC centre: CCP−IMG
Eosinophil differential count
IMPC centre: ICS
Eosinophil differential count
IMPC centre: MRC Harwell
Eosinophil differential count
IMPC centre: RBRC
Eosinophil differential count
IMPC centre: TCP
Eosinophil differential count
IMPC centre: UC Davis
Eosinophil differential count

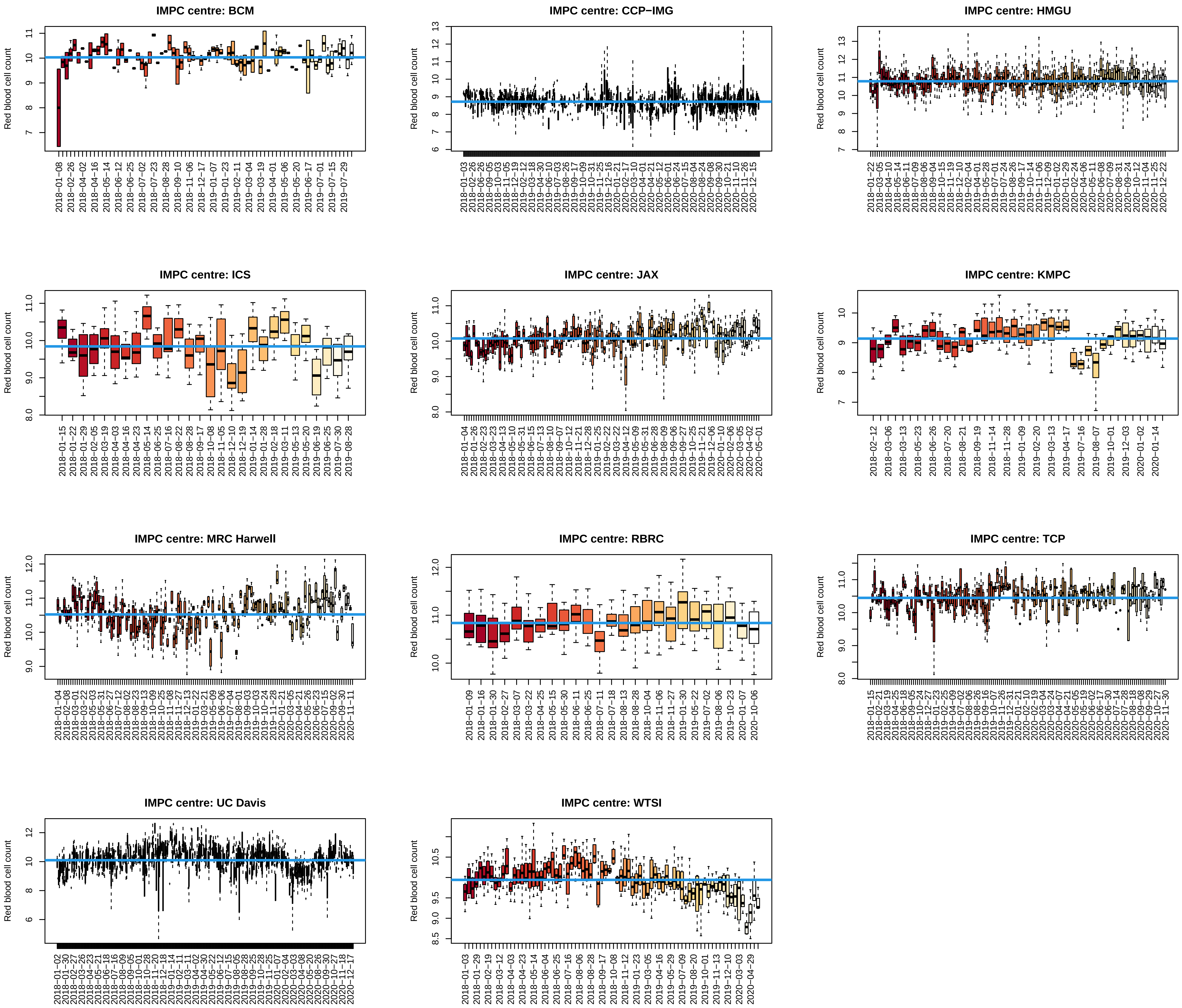
IMPC centre: BCM
IMPC centre: CCP−IMG
IMPC centre: HMGU
IMPC centre: ICS
IMPC centre: JAX
IMPC centre: KMPC
IMPC centre: MRC Harwell
IMPC centre: RBRC
IMPC centre: TCP
IMPC centre: UC Davis
IMPC centre: WTSI
Red blood cell count

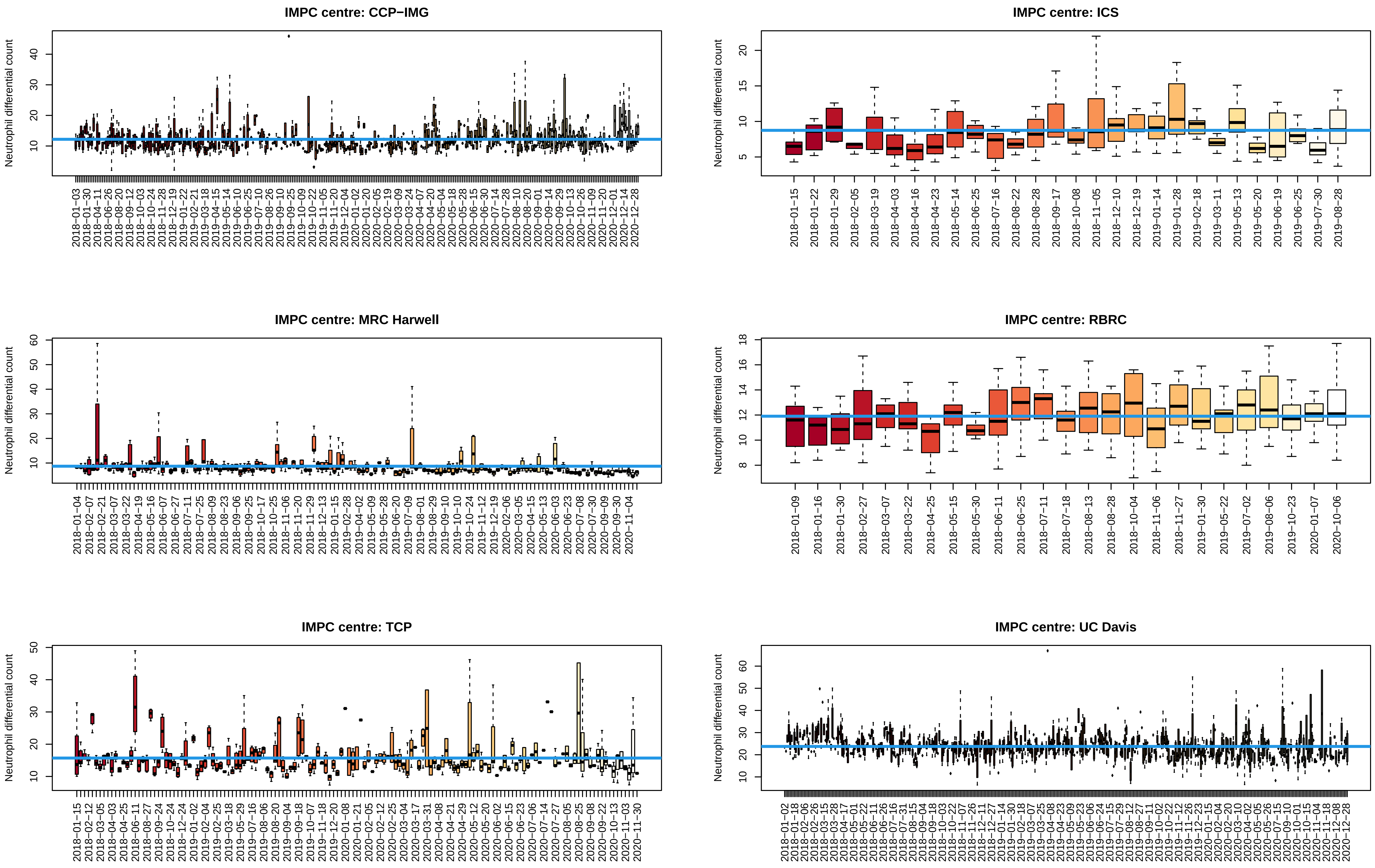
IMPC centre: CCP−IMG
IMPC centre: ICS
IMPC centre: MRC Harwell
IMPC centre: RBRC
IMPC centre: TCP
IMPC centre: UC Davis
Neutrophil differential count

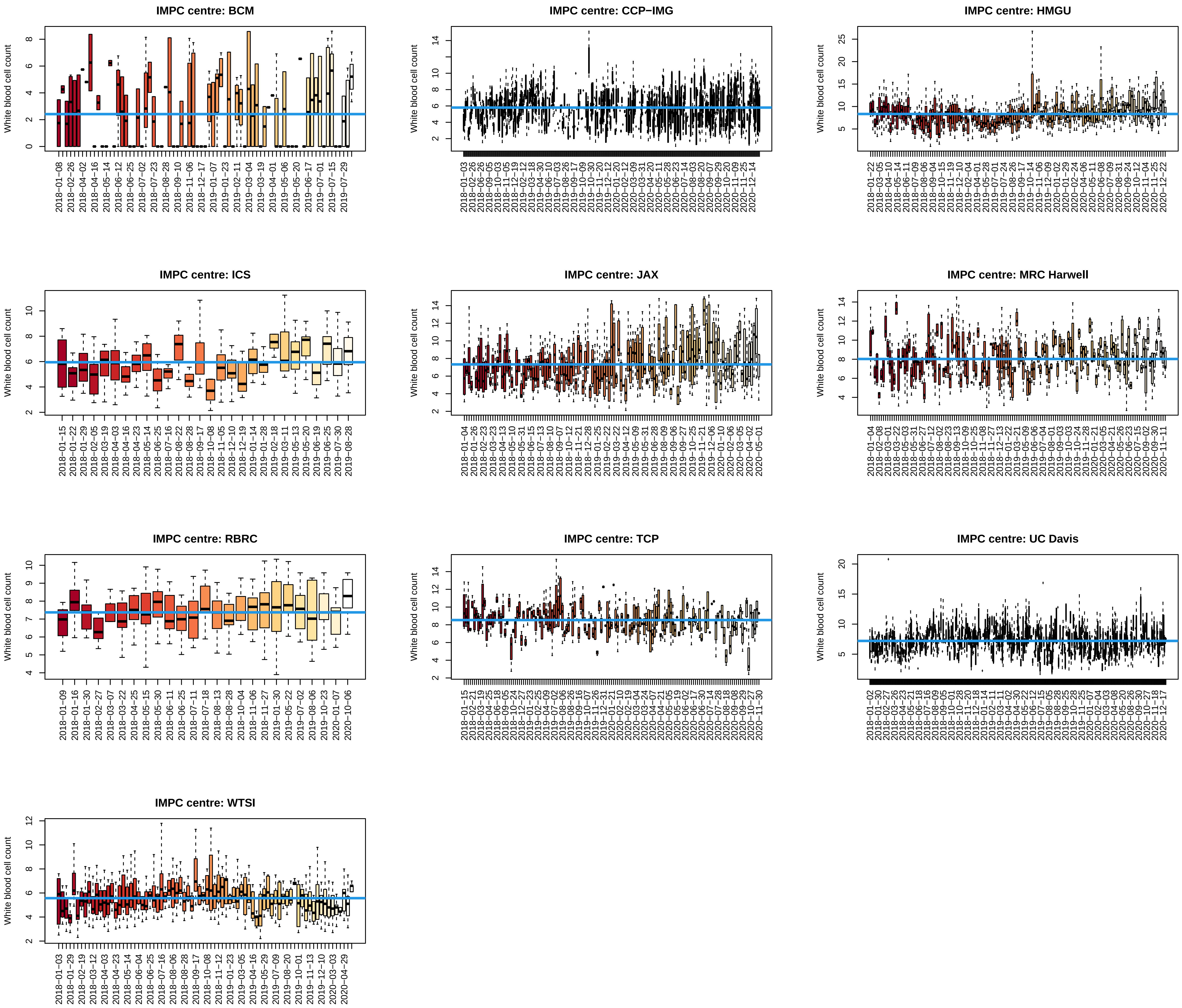
IMPC centre: BCM
IMPC centre: CCP−IMG
IMPC centre: HMGU
IMPC centre: ICS
IMPC centre: JAX
IMPC centre: MRC Harwell
IMPC centre: RBRC
IMPC centre: TCP
IMPC centre: UC Davis
IMPC centre: WTSI
White blood cell count

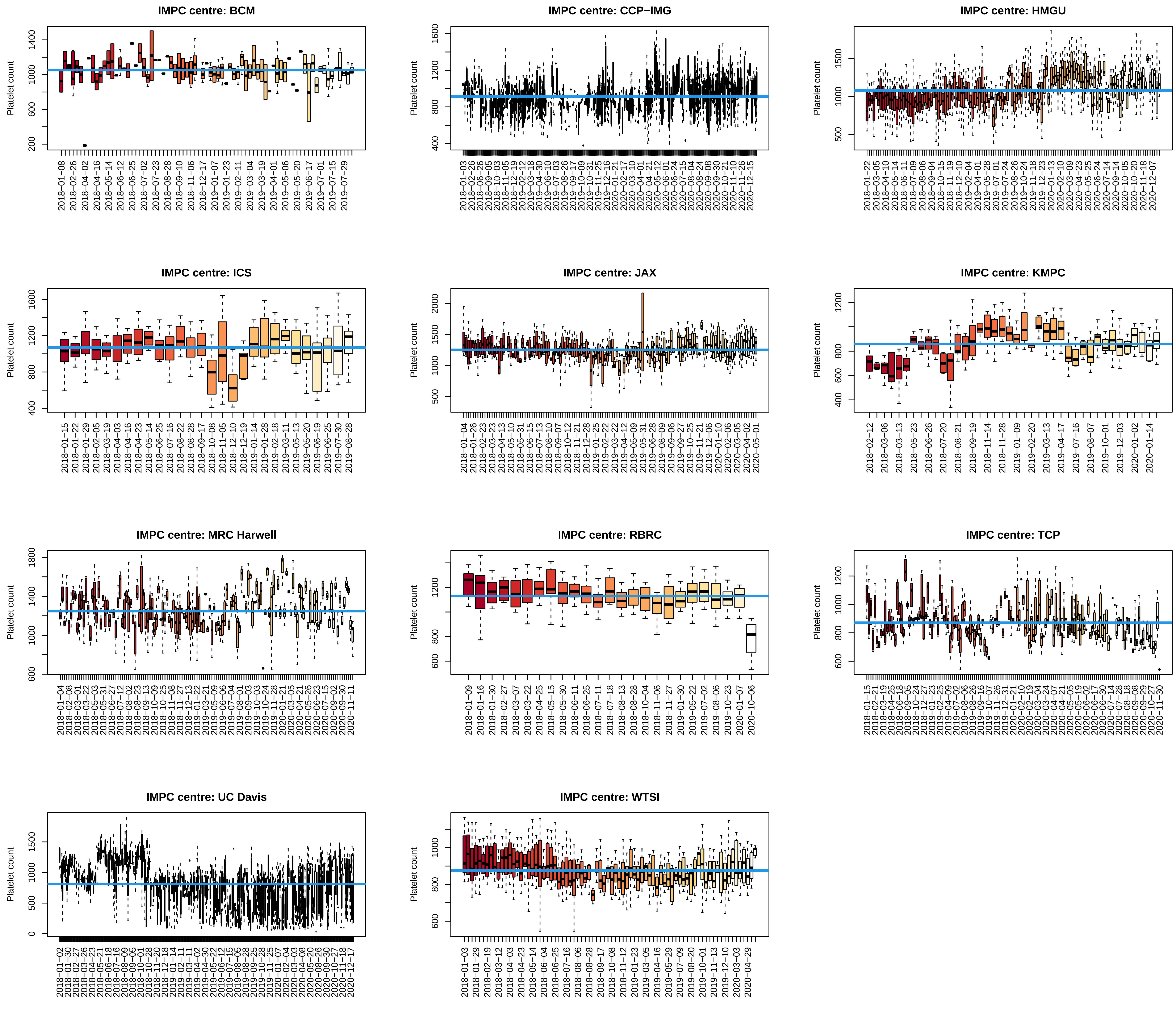
IMPC centre: BCM
IMPC centre: CCP−IMG
IMPC centre: HMGU
IMPC centre: ICS
IMPC centre: JAX
IMPC centre: KMPC
IMPC centre: MRC Harwell
IMPC centre: RBRC
IMPC centre: TCP
IMPC centre: UC Davis
IMPC centre: WTSI
Platelet count

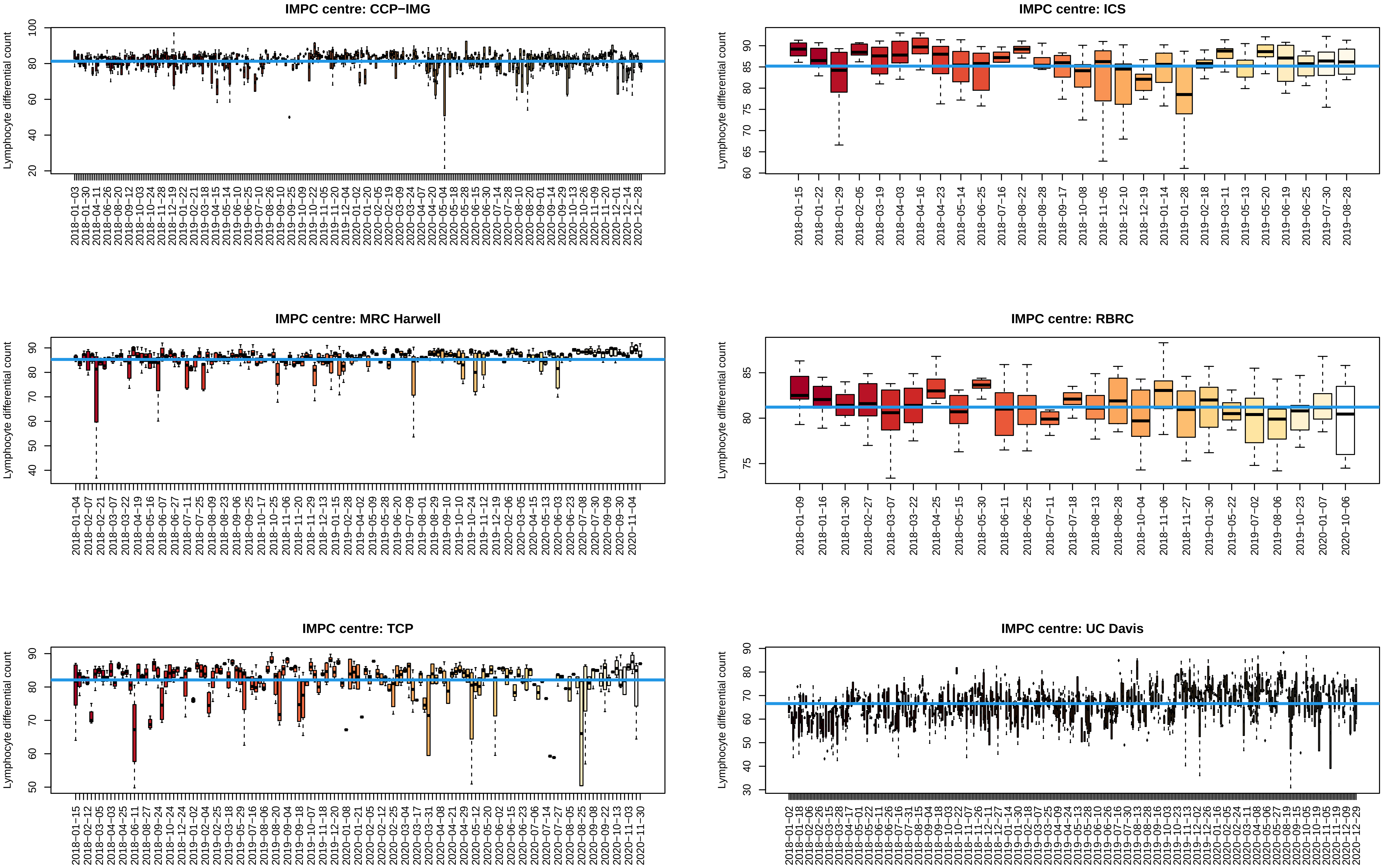
IMPC centre: CCP−IMG
IMPC centre: ICS
IMPC centre: MRC Harwell
IMPC centre: RBRC
IMPC centre: TCP
IMPC centre: UC Davis
Lymphocyte differential count

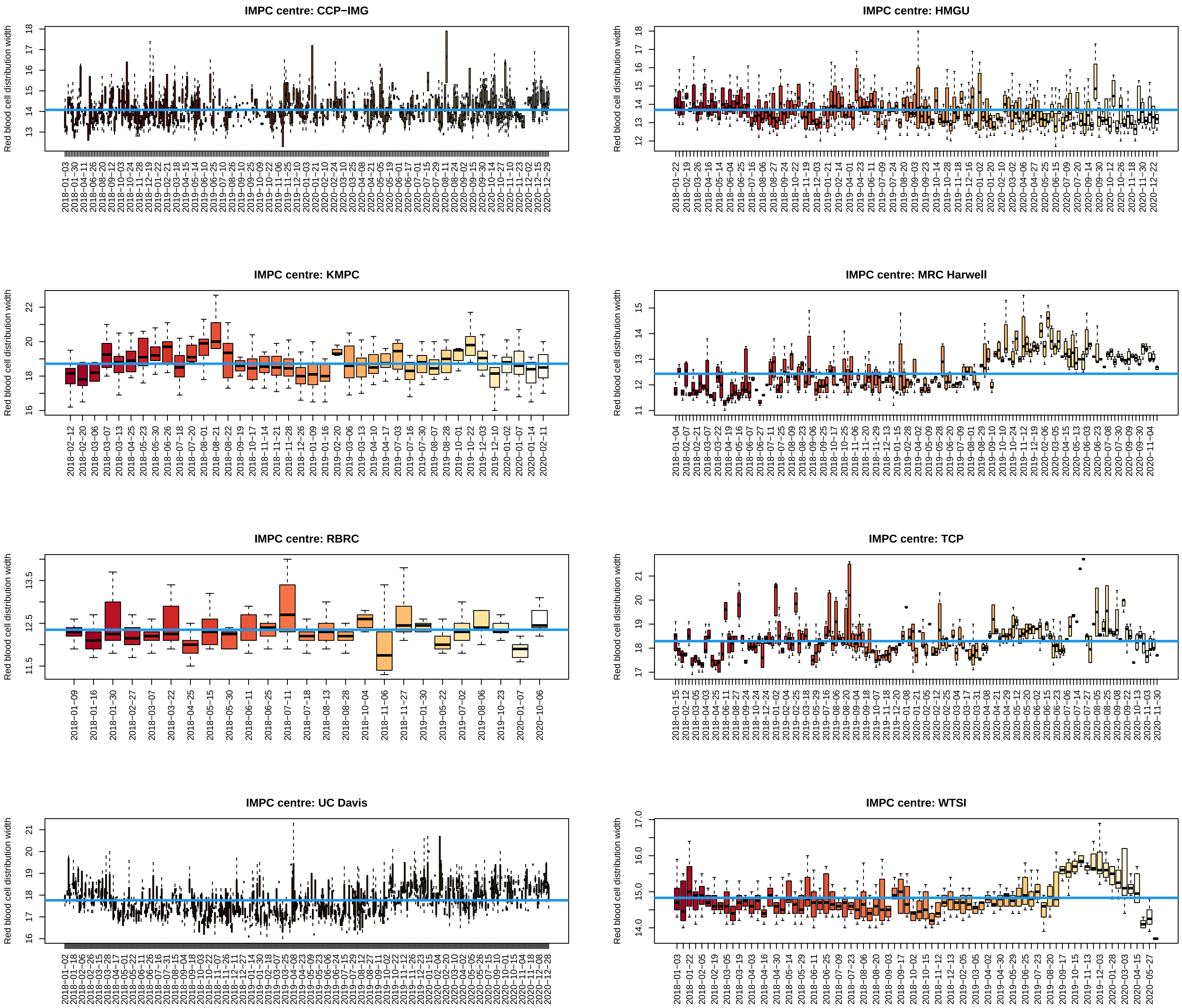

IMPC centre: CCP−IMG
IMPC centre: HMGU
IMPC centre: KMPC
IMPC centre: MRC Harwell
IMPC centre: RBRC
IMPC centre: TCP
IMPC centre: UC Davis
IMPC centre: WTSI
Red blood cell distribution width

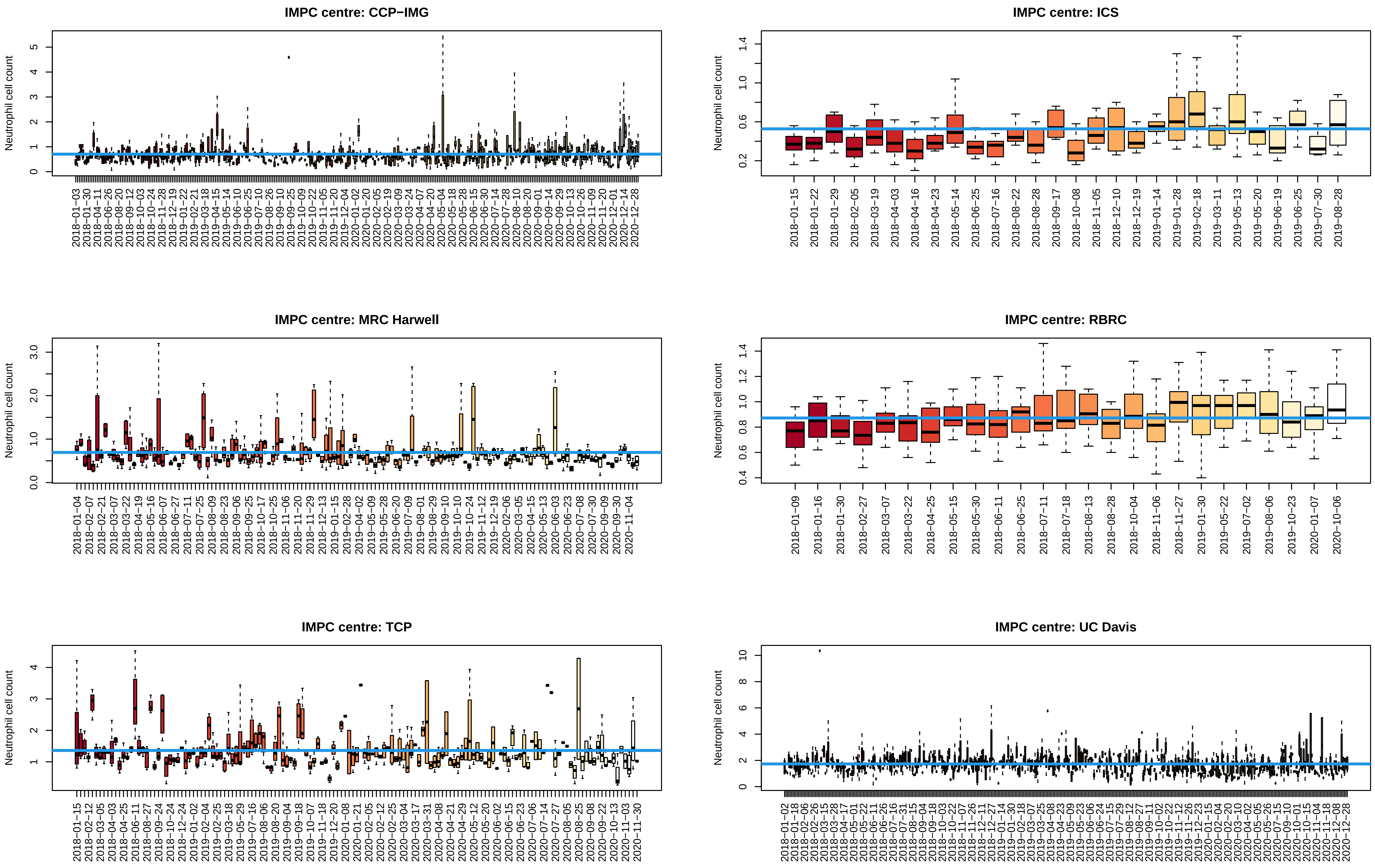
IMPC centre: CCP−IMG
IMPC centre: ICS
IMPC centre: MRC Harwell
IMPC centre: RBRC
IMPC centre: TCP
IMPC centre: UC Davis
Neutrophil cell count

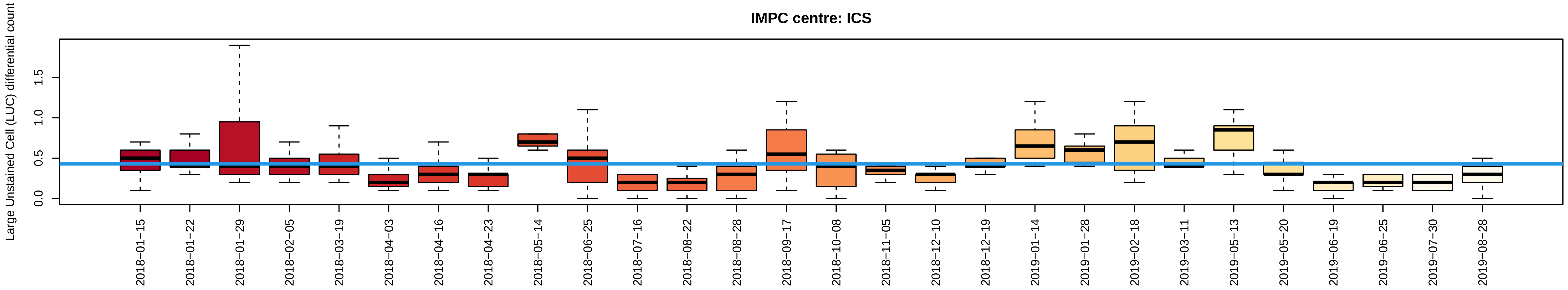
IMPC centre: ICS
Large Unstained Cell (LUC) differential count
0.0
0.5
1.0
1.5
2018-01-15
2018-01-22
2018-01-29
2018-02-05
2018-03-19
2018-04-03
2018-04-16
2018-04-23
2018-05-14
2018-06-25
2018-07-16
2018-08-22
2018-08-28
2018-09-17
2018-10-08
2018-11-05
2018-12-10
2018-12-19
2019-01-14
2019-01-28
2019-02-18
2019-03-11
2019-05-13
2019-05-20
2019-06-19
2019-06-25
2019-07-30
2019-08-28

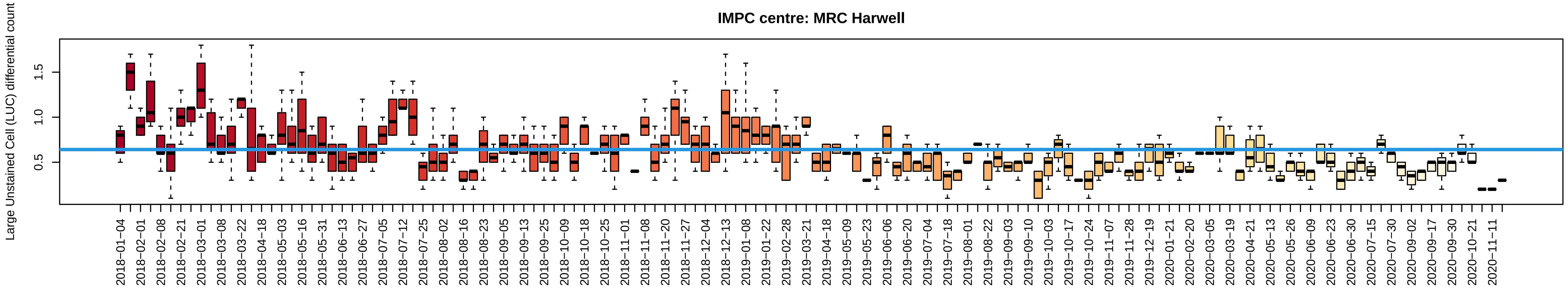
IMPC centre: MRC Harwell
Large Unstained Cell (LUC) differential count
0.5
1.0
1.5
2018-01-04
2018-02-01
2018-02-08
2018-02-21
2018-03-01
2018-03-08
2018-03-22
2018-04-18
2018-05-03
2018-05-16
2018-05-31
2018-06-13
2018-06-27
2018-07-05
2018-07-12
2018-07-25
2018-08-02
2018-08-16
2018-08-23
2018-09-05
2018-09-13
2018-09-25
2018-10-09
2018-10-18
2018-10-25
2018-11-01
2018-11-08
2018-11-20
2018-11-27
2018-12-04
2018-12-13
2019-01-08
2019-01-22
2019-02-28
2019-03-21
2019-04-18
2019-05-09
2019-05-23
2019-06-06
2019-06-20
2019-07-04
2019-07-18
2019-08-01
2019-08-22
2019-09-03
2019-09-10
2019-10-03
2019-10-17
2019-10-24
2019-11-07
2019-11-28
2019-12-19
2020-01-21
2020-02-20
2020-03-05
2020-03-19
2020-04-21
2020-05-13
2020-05-26
2020-06-09
2020-06-23
2020-06-30
2020-07-15
2020-07-30
2020-09-02
2020-09-17
2020-09-30
2020-10-21
2020-11-11

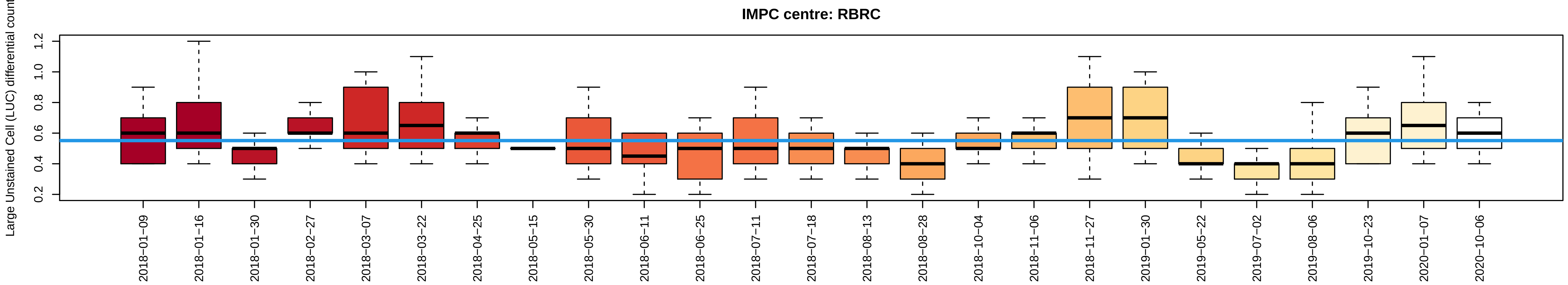
IMPC centre: RBRC
Large Unstained Cell (LUC) differential count
0.2
0.4
0.6
0.8
1.0
1.2
2018-01-09
2018-01-16
2018-01-30
2018-02-27
2018-03-07
2018-03-22
2018-04-25
2018-05-15
2018-05-30
2018-06-11
2018-06-25
2018-07-11
2018-07-18
2018-08-13
2018-08-28
2018-10-04
2018-11-06
2018-11-27
2019-01-30
2019-05-22
2019-07-02
2019-08-06
2019-10-23
2020-01-07
2020-10-06

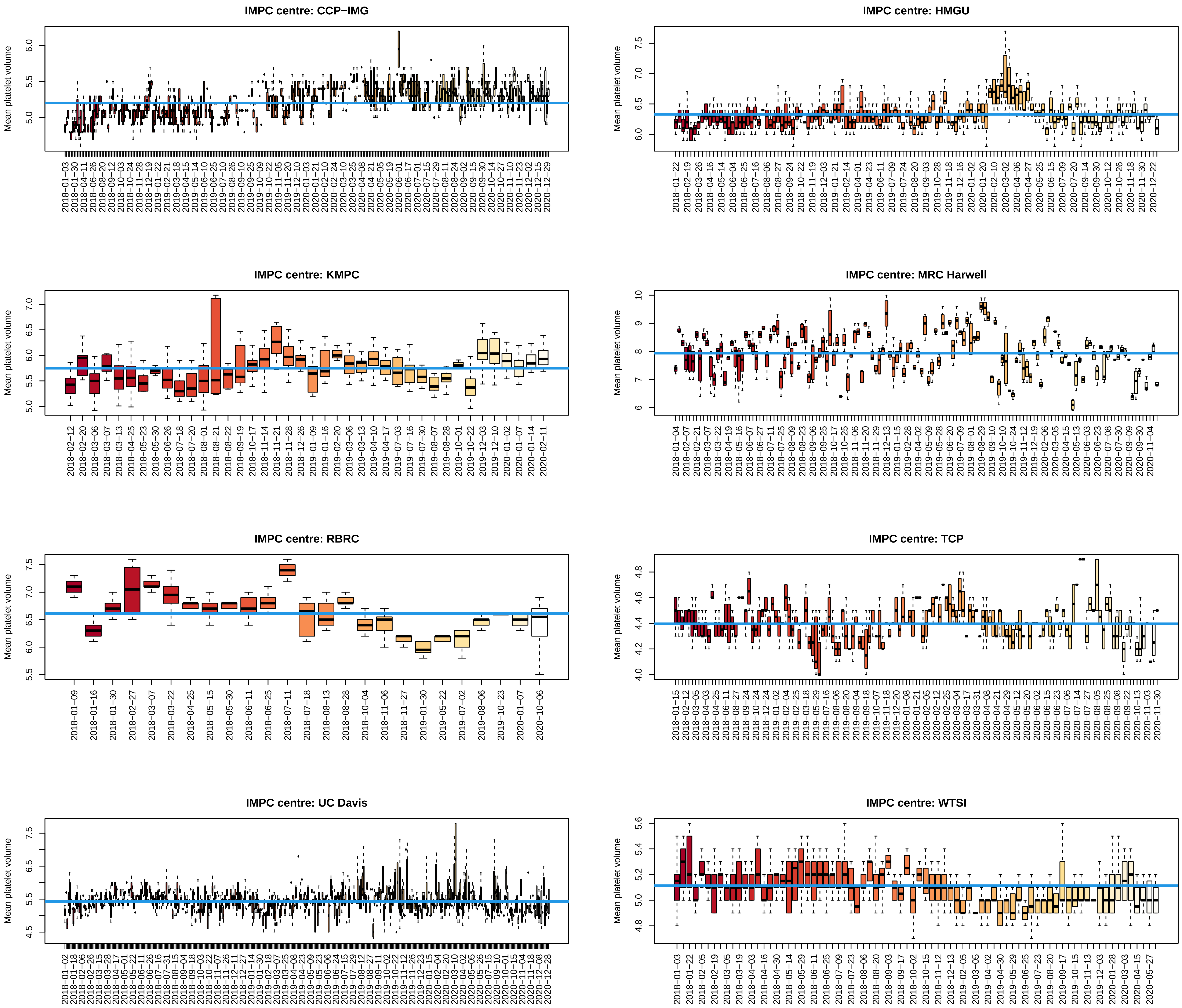
IMPC centre: CCP−IMG
Mean platelet volume
5.0
5.5
6.0
IMPC centre: HMGU
Mean platelet volume
6.0
6.5
7.0
7.5
IMPC centre: KMPC
Mean platelet volume
5.0
5.5
6.0
6.5
7.0
IMPC centre: MRC Harwell
Mean platelet volume
6
7
8
9
10
IMPC centre: RBRC
Mean platelet volume
5.5
6.0
6.5
7.0
7.5
IMPC centre: TCP
Mean platelet volume
4.0
4.2
4.4
4.6
4.8
IMPC centre: UC Davis
Mean platelet volume
4.5
5.5
6.5
7.5
IMPC centre: WTSI
Mean platelet volume
4.8
5.0
5.2
5.4
5.6
